# ROS-producing nanomaterial engineered from Cu(I) complexes with P_2_N_2_-ligands for cancer cells treating

**DOI:** 10.1186/s11671-023-03912-7

**Published:** 2023-10-30

**Authors:** Bulat A. Faizullin, Irina R. Dayanova, Alexey V. Kurenkov, Aidar T. Gubaidullin, Alina F. Saifina, Irek R. Nizameev, Kirill V. Kholin, Mikhail N. Khrizanforov, Aisylu R. Sirazieva, Igor A. Litvinov, Alexandra D. Voloshina, Anna P. Lyubina, Guzel V. Sibgatullina, Dmitry V. Samigullin, Elvira I. Musina, Igor D. Strelnik, Andrey A. Karasik, Asiya R. Mustafina

**Affiliations:** 1 Arbuzov Institute of Organic and Physical Chemistry, FRC Kazan Scientific Center of RAS, 8 Arbuzov Str., Kazan, Russia 420088; 2 Department of Physics, Kazan National Research Technological University, 68 Karl Marx Str., Kazan, Russia 420015; 3 Department of Nanotechnology in Electronics, Kazan National Research Technical University Named After A.N. Tupolev-KAI, 10 K. Marx Street, Kazan, Russia 420111; 4 Aleksander Butlerov Institute of Chemistry, Kazan Federal University, 1/29 Lobachevski Str., Kazan, Russia 420008; 5 Kazan Institute of Biochemistry and Biophysics, FRC Kazan Scientific Center of RAS, 2/31 Lobachevski Str., Kazan, Russia 420111; 6 Institute for Radio-Electronics and Telecommunications, Kazan National Research Technical University Named After A.N. Tupolev-KAI, 10 K. Marx Street, Kazan, Russia 420111

**Keywords:** Copper(i) complex, Hydrophilic nanoparticles, Luminescence, ROS generation, Chemodynamic therapy

## Abstract

The work presents core–shell nanoparticles (NPs) built from the novel Cu(I) complexes with cyclic P_2_N_2_-ligands (1,5-diaza-3,7-diphosphacyclooctanes) that can visualize their entry into cancer and normal cells using a luminescent signal and treat cells by self-enhancing generation of reactive oxygen species (ROS). Variation of P- and N-substituents in the series of P_2_N_2_-ligands allows structure optimization of the Cu(I) complexes for the formation of the luminescent NPs with high chemical stability. The non-covalent modification of the NPs with triblock copolymer F-127 provides their high colloidal stability, followed by efficient cell internalization of the NPs visualized by their blue (⁓450 nm) luminescence. The cytotoxic effects of the NPs toward the normal and some of cancer cells are significantly lower than those of the corresponding molecular complexes, which correlates with the chemical stability of the NPs in the solutions. The ability of the NPs to self-enhanced and H_2_O_2_-induced ROS generation is demonstrated in solutions and intracellular space by means of the standard electron spin resonance (ESR) and fluorescence techniques correspondingly. The anticancer specificity of the NPs toward HuTu 80 cancer cells and the apoptotic cell death pathway correlate with the intracellular level of ROS, which agrees well with the self-enhancing ROS generation of the NPs. The enhanced level of ROS revealed in HuTu 80 cells incubated with the NPs can be associated with the significant level of their mitochondrial localization.

**Supplementary Information:**

The online version contains supplementary material available at 10.1186/s11671-023-03912-7.

## Introduction

Cu(I) complexes have gained great attention in recent decades due to their ability to catalyze the Fenton-like reactions leading to the ROS generation, since the latter allows a cell treating through an oxidation stress or the so-called chemodynamic therapy (CDT) [1–15]. In most cases, the ROS generation via the Fenton-like reactions is triggered by hydrogen peroxide or the gentle temperature increase without light irradiation [1–3]. However, developing of Cu(I) complex species exhibiting self-boosting ROS generation is very attractive, but challenging task. Summarizing literature data, the main requirements to Cu(I) complexes for their use as CDT agents are: (1) enough water solubility; (2) negligible oxidative degradation in aqueous environment; (3) high level of ROS generated in a self-boosting mode or triggered by endogenous H_2_O_2_; (4) significant cellular uptake; and (5) luminescent properties allowing to reveal a cell internalization and intracellular trafficking of the complexes. Literature data introduce significant success in the developing of the ligand environment of Cu(I) ions fitting to the above-mentioned requirements. However, structure optimization on both molecular and nanolevels is a powerful tool to solve the above-mentioned problems. In particular, the key problems concerning catalytic activity in the Fenton-like reactions and luminescence can be solved at the molecular level only, while such properties as stability to oxidative degradation, enough water solubility and cellular uptake can be achieved in a framework of the well-known nanoparticulate approach. The present work is aimed at highlighting an impact of molecular structure of the Cu(I) complexes on their catalytic activity in the Fenton-like reactions, as well as to demonstrate the nanoparticulate approach as a tool to enhance the biocompatibility and uptake of the complexes by cells.

The cyclic P_2_N_2_-ligands (1,5-diaza-3,7-diphosphacyclooctanes) should be noted as a convenient platform for a wide structural diversity of complexes with d^10^ metal ions, which can be implemented by introducing various substituents to the phosphorus and nitrogen atoms [16, 17]. This makes the ligands an attractive platform for the design of various coordination compounds with tunable properties, since the P- and N-substituents dictate the properties of the complexes due to their involvement into the excitation and emission electronic transitions. The structure of the substituents is also of great impact on the solubility of the complexes. Moreover, the stability of Cu(I) P,P-chelate complexes based on this type of ligands is significantly increased both due to the chelating effect of two ligands and due to shielding of copper(I) complexes by ring N-substituents, which was demonstrated for complexes in solid state and in various organic solvents [18]. Thus, the variation of P- and N-substituents in the series of P_2_N_2_-ligands is aimed at optimizing the structure of their Cu(I) complexes for their suitability as building blocks of hydrophilic luminescent NPs with high chemical and colloidal stability.

The recent reports demonstrate great potential of Cu(I)-based nanomaterial fabricated from the Cu(I) complexes in developing of the CDT-based routes for anticancer treatment [9, 13, 19–25]. It is worth noting the easy way to enhance a hydrophilicity of nanomaterial by its non-covalent surface modification [22], while a water solubility of molecular complexes requires complicated synthetic modification of the ligands. The chemical stability of Cu(I)-based nanomaterial being greater than that of molecular complexes prerequisites the stable in time luminescence in the intracellular environment [9] and minimal side effect under the in vivo application [21]. The so-called cuproptosis is one of the causes of increased non-apoptotic cytotoxicity [26]. It follows from this that the incorporation of Cu(I) complexes into a nanomaterial can become a reliable tool for increasing the contribution of the apoptotic cell death mechanism compared to non-apoptotic one. Thus, the present work represents the easy one-pot synthetic route allowing transformation of the Cu(I) complexes into hydrophilic luminescent NPs through the stimuli-induced aggregation of the complexes with following non-covalent surface modification by the well-known nontoxic triblock copolymer F-127, which has been already successfully applied in colloid stabilization of the nanoscale Cu(I) complexes [9, 22]. The represented results reveal the structural features of the ligands responsible for the formation of the luminescent NPs. The structural features of the complexes facilitating their aggregation into nanosized colloidal species in aqueous solutions are also discussed.

The specific redox behavior of complexes is the already known factor affecting their catalytic activity in Fenton-like reactions [13, 15, 27]. However, the impact of the electrochemical parameters of Cu(I) complexes on their catalytic activity in Fenton-like reactions is not well recognized. Thus, the electrochemical behavior of the synthesized complexes in the solid state and in solutions is presented for further correlation with their ability to generate ROS in aqueous solutions after their transformation into hydrophilic NPs. The tailoring of the Cu(I)-based NPs by the hydrophilic shells is demonstrated as the tool to enhance their cellular uptake behavior visualized by the fluorescent microscopy techniques. The cytotoxicity values determined for the different cancer and normal cell lines indicate the selective anticancer specificity in good agreement with the self-boosting ROS generation of the Cu(I)-based NPs.

## Materials and methods

The details of the Materials and Methods section regarding used materials, synthesis and characterization of ligands and complexes, synthesis of NPs, general methods, crystallography (CCDC 2270682 (complex **1**), 2270680 (complex **3**)) and descriptions of biological methods are given in Supplementary Material.

## Results and discussion

### Synthesis of ligands and complexes

**Scheme 1:**
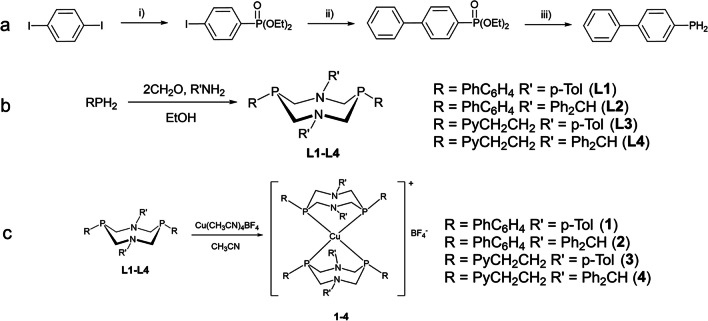
**a** Synthesis of biphenylphosphine: i) P(OEt)_3_, NiCl_2_ (anhydrous, 5 mol %), Δ160 °C; ii) phenylboronic acid, (Ph_3_P)_4_Pd (5 mol %), NaHCO_3_, toluene; and iii) LiAlH_4_, Et_2_O. **b** Synthesis of P_2_N_2_-ligands **L1**-**L4**. **c** Synthesis of complexes **1-4**

Initial biphenylphosphine was obtained by the phosphorylation of 1,4-diodobenzene followed with the reaction of obtained 4-iodophenyl-O,O-diethylphosphonate [28] and phenylboronic acid (Suzuki cross-coupling reaction) and the reduction of biphenylphosphonate by LiAlH_4_ (Scheme 1a).

Reaction of the biphenylphosphine with two equivalents of paraform and *p*-toluidine or benzhydrylamine led to the formation of the 1,5-diaza-3,7-diphosphacyclooctanes (P_2_N_2_-ligands **L1** and **L2**) with the yields of 50–60% (Scheme 1b). P_2_N_2_-ligands **L3** and **L4** were obtained according to synthetic procedures described earlier [29, 30]. Interaction of the ligands **L1**-**L4** with Cu(CH_3_CN)_4_BF_4_ in acetonitrile led to the formation of the bis-P,P-chelate complexes **1-4** with the quantitative yields (Scheme 1c).

In the NMR ^31^P spectra (Figs. S1–S6), the signals of complexes (ca. − 14 to − 20 ppm) strongly downfield shifted relative to the signals of the free ligands (− 50 to − 60 ppm), which corresponds to the formation of the P,P-chelate cycle. In the ESI mass spectra (Figs. S7–S10), only one peak corresponding to the [2L + Cu]^+^ was registered.

**Fig. 1:**
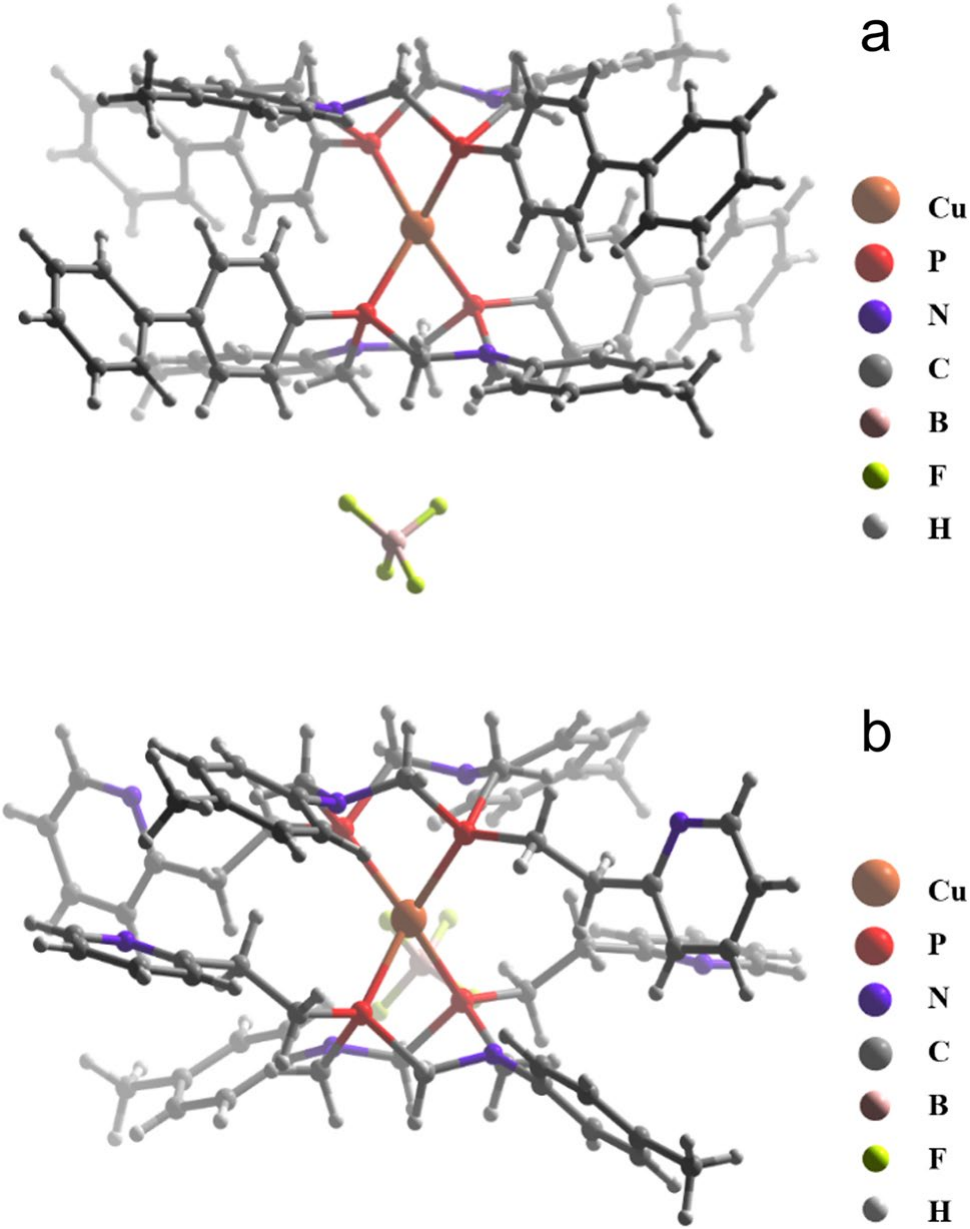
XRD molecular structure of complexes **1** (**a**) and **3** (**b**)

The structures of complexes **1** and **3** in the solid state have been confirmed by the XRD analysis (Fig. 1).

In both complexes, Cu(I) has a tetrahedral coordination geometry of ligand environment formed by four P-Cu bonds of two ligands. The ligands in the complexes exist in “chair-chair” conformations with flattened C–N–C parts. This conformation is slightly unusual for this type of ligands, which derives from the movement of the N-substituents from axial to equatorial position due to the steric hindrance of the substituents of the opposite ligands. The bite angles values are 81.24(5)° for complex **1** and 84.60(4)° and 85.64(4)° for complex **3**, which is similar to the other chelate complexes of P_2_N_2_-ligands [18, 31–33]. It is also worth noting that BF_4_^–^ anions form F–H interactions with the PCH_2_N-moieties of aminomethyl cycles in both complexes. (F–H distances are 2.27 and 2.51 for complexes **1** and **3**, respectively.)

### Synthesis of Cu(I) complexes-based NPs

**Fig. 2:**
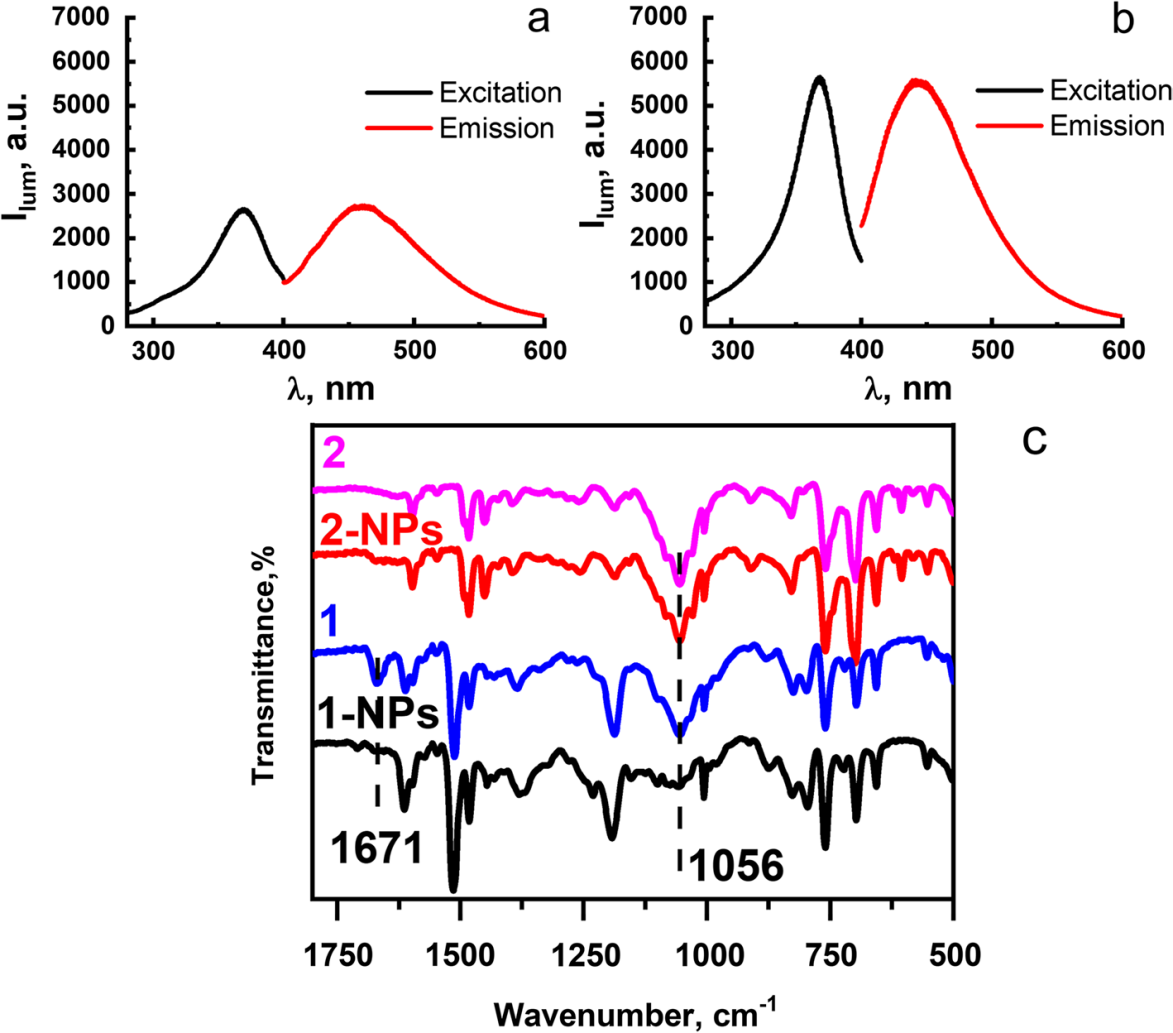
Excitation and emission spectra of F-127-**1** (**a**) and F-127-**2** (**b**) NPs. (**c**) FTIR spectra of dried complex **1**- and complex **2**-based NPs and corresponding powder samples of complexes **1** and **2**

The significant solubility of complexes **1**-**4** (Scheme 1c) in DMF and poor solubility in water triggers their aggregation in the aqueous–organic solutions. Bulky aromatic substituents at the P- and N-atoms of macrocyclic P_2_N_2_-ligands (Scheme 1c) provide sufficient shielding of Cu^+^ centers, which protects complexes **1**-**4** from hydration transformations in aqueous–organic solutions. As a result of aggregation, NPs are formed, which size and aggregative behavior can be controlled by optimizing of synthesis conditions. Varying the ratio of the volumes of the aqueous and organic phases, the concentration of the salting out agent (NaCl) and the triblock copolymer (F-127) makes it possible to optimize synthetic conditions (for more details see the SI). The presence of F-127 is aimed at hydrophilic coating of the formed NPs in order to prevent their aggregation. The analysis of the luminescent and colloidal properties of the produced aggregates reveals an impact of the aromatic substituents at phosphorus atoms on their luminescent (Figs. 2 and S11) and colloidal properties (Tables 1 and S1). In particular, complexes **1** and **2** exhibit luminescence in both DMF solutions (Fig. S11) and in aqueous environment after their conversion into NPs, which hereinafter will be designated as F-127-**1**(**2**) (Fig. 2a,b). The poor luminescence of complexes **3** and **4** in DMF solutions and after their reprecipitation in the aqueous-DMF solutions (Fig. S11) along with low colloidal stability of complex **3**-based NPs (Table S1) are the reasons for excluding these complexes from further studies. The greater vibrational and rotational mobility of the pyridylethyl substituents onto P-atoms in complexes **3**, **4** *vs* complexes **1**, **2** possessing rigidly fixed biphenyl substituents onto P-atoms agrees well with the aforesaid difference in their spectral behavior. The difference in the maximum of the emission bands for the complexes in the DMF solutions (Fig. S11) and in F-127-**1**(**2**) aqueous colloids (Fig. 2a,b) may be due to the fact that the emission of Cu(I) complexes with cyclic P_2_N_2_-ligands is contributed by both singlet and triplet excited states and their contribution depends on many external factors, such as temperature, solvent and packing mode of the complexes [18]. However, the estimation of the exact mechanisms of the complexes’ emission in DMF solutions and in the nanoparticulate form lies out of the present work scope.

**Table 1:** Average (*d*_av_) and evaluated through the size distribution by number (*d*_num_) diameter values measured by DLS, polydispersity indices (PDI) and electrokinetic potentials (ζ) of F-127-**1**(**2**) NPs in pure water and buffered solutions

	*d*_av_, nm	*d*_num_, nm	PDI	ζ, mV
F-127-**1**	156 ± 4	139 ± 3	0.090 ± 0.016	+ 27 ± 7
F-127-1*	143 ± 1	124 ± 5	0.106 ± 0.014	+ 12 ± 7
F-127-1**	144 ± 1	119 ± 9	0.102 ± 0.006	–4 ± 6
F-127-**2**	173 ± 4	155 ± 9	0.064 ± 0.022	+ 25 ± 7
F-127-2*	154 ± 1	135 ± 10	0.077 ± 0.018	+ 16 ± 7
F-127-2**	156 ± 1	135 ± 6	0.091 ± 0.014	–1 ± 9

*pH 4.0

**pH 7.0

The Cu/P molar ratios calculated from the ICP-OES data are close to 1:4, which argues for the chemical stability of complexes **1** and **2** when they are converted into the aqueous colloids (Table S2). The safe transition of complex **2** into the colloidal phase during its reprecipitation under solvent exchange conditions is evidenced by a comparative analysis of the FTIR data measured for NPs and powder samples of complexes (Figs. 2c and S12). A comparative analysis of the FTIR data for complex **1** in the colloidal and initial powder samples reveals the absence of the band at 1671 cm^–1^, which most probably corresponds to residual amounts of DMF in the initial compound (Fig. S13). Besides, whereas the spectrum of the initial powder sample contains the strong band at 1056 cm^–1^ associated with the BF_4_^–^, the intensity of this band in the spectrum of the colloidal sample is significantly decreased (Fig. 2c). This can be explained by the counter-ion exchange under the reprecipitation of complex **1** in the aqueous-DMF solutions of sodium chloride. Meanwhile, the FTIR data for dried complex **2**-based NPs reveal the insignificant difference from those of the powder sample of the initial complex **2** with remaining unchanged the band arisen from BF_4_^–^ (Fig. 2c).

PXRD measurements were performed to compare the phase state of dried F-127-**1**(**2**) with that of the initial powder samples. In contrast to the amorphous nature of complex **2**, which was revealed in both colloidal and initial powder samples, the initial powder of complex **1** is crystalline, and its transition to the colloidal phase is accompanied by a change in the XRD (Fig. S12). The crystalline nature of the powder samples of complex **1** is based on their supramolecular assembly with the help of counter-ions (Fig. S14). Thus, the pronounced changes in the supramolecular packing of complex **1** during its conversion into the colloidal phase can be explained by the counter-ion exchange revealed from the FTIR data (Fig. 2c). The PXRD pattern of dried complex **2**-based NPs indicates a disordered supramolecular packing of complex **2** similar with that to the initial powder sample (Fig. S12), which agrees well with the insignificant counter-ion exchange during the formation of F-127-**2** revealed from the FTIR data (Fig. 2c).

**Fig. 3:**
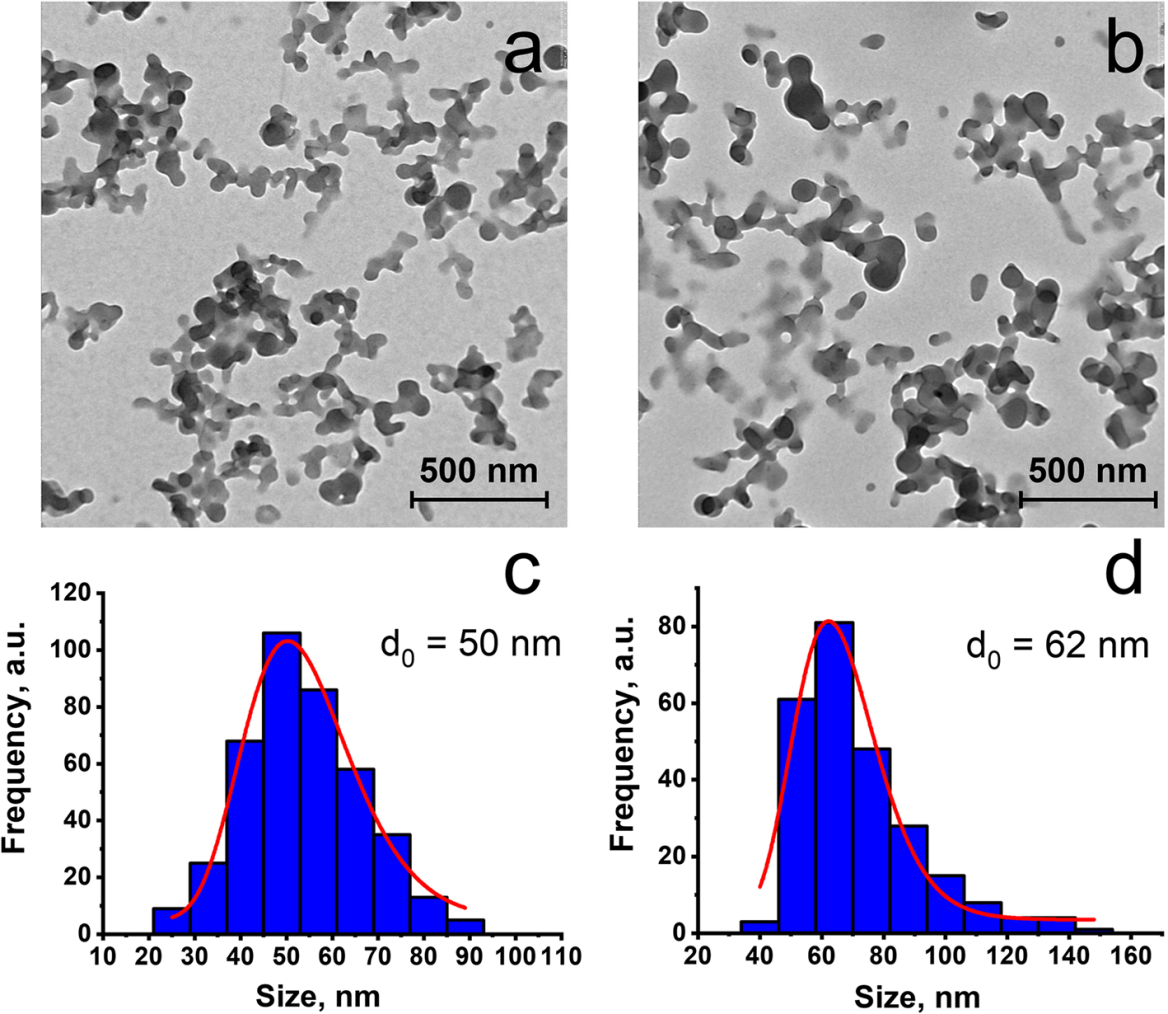
TEM images (**a**, **b**) and the size distribution histograms (**c**, **d**) of F-127-**1** (**a**, **c**) and F-127-**2** (**b**, **d**) NPs

The TEM images of the dried colloids reveal the spherical NPs with blurred boundaries, which agrees well with their amorphous nature (Fig. 3). The sizes of the NPs are deviating within 40–70 nm and 50–80 for the dried F-127-**1** and F-127-**2**, respectively.

### Luminescence of the Cu(I) complexes-based NPs. Chemical and colloidal stability

F-127-**1**(**2**) NPs in aqueous dispersions manifest itself as blue emitting luminophores (Fig. 2a,b) with high size uniformity and colloidal stability in aqueous dispersions (Table 1), while an incontrollable aggregation of the reprecipitated complexes **1** and **2** is observed in the absence of F-127. The colloidal stability of F-127-**1**(**2**) derives from the formation of hydrophilic F-127-based exterior layer onto the aggregated complexes **1** and **2**, and thus, the aggregation behavior of the NPs remains unchanged in the buffer solutions (Table 1). The quantum yield (QY) values of complexes **1** and **2** in the form of F-127-**1**(**2**) NPs are 3.5% and 5.4%, respectively. These values are close to those of complexes **1** and **2** in the solid state, which are 4.1% and 6.1%, correspondingly. Such values are typical for the Cu(I) complexes with P_2_N_2_-ligands [18].

The luminescence of F-127-**1**(**2**) NPs allows to control their chemical stability within the incubation process and in the intracellular environment conditions. Thus, the luminescence of F-127-**1**(**2**) was monitored over the time, after the gentle heating to physiological temperatures and in the solutions of hydrogen peroxide and glutathione (GSH) at pH 7.0. The measurements were also performed at pH 4.0, and the acidified conditions were chosen to simulate the increased acidity of the lysosomal microenvironment, since a cell internalization of NPs commonly undergoes through a lysosomal pathway. The emission bands of F-127-**1**(**2**) remain practically unchanged after the storage of the NPs for 7 days in a buffered solution at 7.0 (Fig. S15). The colloidal behavior of F-127-**1**(**2**) is also unchanged after their storage for 7 days in pure water and in the buffer solution (Table S3). It is worth noting that storing F-127-**1**(**2**) NPs for 7 days in a buffer solution at pH 4.0 is accompanied by a slight decrease in their luminescence. This is an argument in favor of insignificant degradation of F-127-**1**(**2**) NPs even under acidified conditions of the lysosomal environment.

**Fig. 4:**
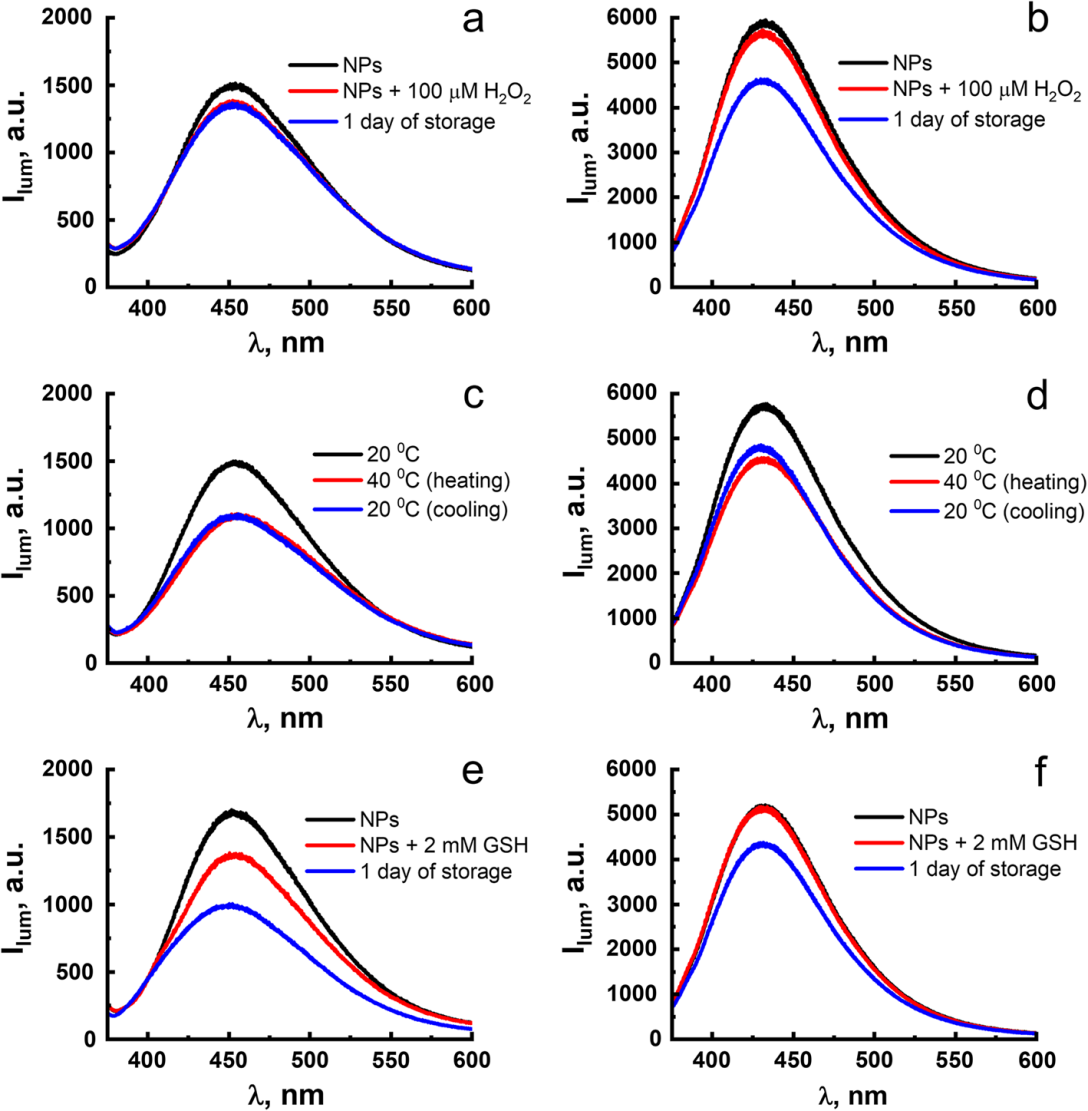
Luminescence spectra of F-127-**1** (**a**, **c**, **e**) and F-127-**2** (**b**, **d**, **f**) NPs in the presence of H_2_O_2_ (**a**, **b**), at different temperatures (**c**, **d**) and in the presence of GSH at pH 7.0

The luminescence of F-127-**1**(**2**) NPs in a solution of hydrogen peroxide (C = 100 μM) shows minor changes within 15 min after sample preparation, while the changes intensify after storing samples for 24 h (Fig. 4a,b). However, the luminescent response to hydrogen peroxide measured over a 24-h period is somewhat greater for F-127-**2** compared to F-127-**1** (Fig. 4a,b). The colloidal behavior of F-127-**1**(**2**) remains unchanged in the solutions of H_2_O_2_ (Table S3). Thus, the luminescence response of the NPs in the solutions of H_2_O_2_ can be associated with the oxidative transformations of the NPs, which are greater in case of F-127–**2** *vs* F-127–**1** (Fig. 4a, b).

Irreversible quenching of the F-127-**1**(**2**) NPs emission is observed upon heating up to 40 °C, although about 80% of the initial intensity remains unchanged after the heating–cooling cycle (Fig. 4c, d). This allows to predict very small degradation of the NPs upon incubation at 37 °C in cells. The analysis of the colloidal properties of F-127-**1**(**2**) after the heating–cooling cycle and after the storage for one day in the solution of H_2_O_2_ reveals the insignificant changes (Table S4).

The efficient complex formation of both GSH and its oxidized form GSSG with Cu(I) ions can trigger a destruction of the complexes associated with the stripping of Cu(I) ions [34–36]. The detectable luminescent response of F-127-**1** to GSH (C = 2 mM) requires exposure for half an hour, while it becomes more pronounced during the day of storage. Smaller changes in luminescence are observed for F-127-**2** in GSH solutions even during the day of storage (Fig. 4e,f). DLS measurements (Table S3) show that the presence of GSH affects the aggregation of F-127-**1** NPs in GSH solutions, which makes it possible to relate the luminescent response of F-127-**1** to GSH with increased colloidal aggregation.

Thus, the above-mentioned tendencies demonstrate the solvent-induced aggregation of the Cu^+^ complexes as the route for the formation of the emitting NPs. Retention of NPs’ luminescence intensity or its slight decrease in solutions of GSH, H_2_O_2_ and under gentle heating reveals a slight or insignificant chemical degradation of the complexes incorporated into the NPs under these conditions.

### Electrochemical measurements of complexes *1* and *2*

The redox behavior of the complexes **1** and **2** was studied by the cyclic voltammetry in DMF solutions and in the solid state with the use of carbon paste electrode in order to characterize specific redox processes in the solution and solid state. The identification of oxidation and reduction peaks provides valuable information about the stability and reactivity of the copper(I) complexes in the aforesaid states.

**Fig. 5:**
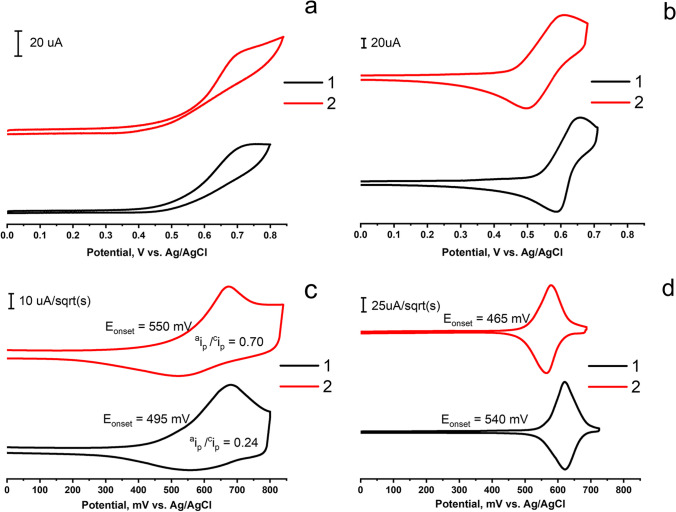
CVs (**a**, **b**) and semi-derivative CVs (**c**, **d**) of oxidation for complexes **1** and **2** in DMF solutions (**a**, **c**) (C = 0.5 mM) and in the solid state (**b**, **d**). Solutions: WE–GC, electrolyte–0.1 M Bu_4_NBF_4_. Solid state: WE–CPE, electrolyte–0.1 M Bu_4_NBF_4_ in CH_3_CN, scan rate–100 mVs^−1^. Potentials vs. Ag/AgCl

Cyclic voltammograms (CVs) measured in the DMF solutions of the complexes in the potential range up to 0.85 V vs. Ag/AgCl are shown in Fig. 5a. The represented CVs demonstrate the single peaks of irreversible oxidation for both complexes (0.75 V for **1** and 0.73 V for **2**). These values are close to those previously introduced for the Cu(I) phosphine complexes [12, 15, 37, 38]. It is important to note that cyclic voltammetry provides valuable information about the redox behavior of electroactive species and the overall electrochemical response. However, relying solely on cyclic voltammetry may overlook important aspects of the electrochemical kinetics and mechanisms occurring at the electrode interface.

The semi-differential CV forms (Fig. 5c) both allow to reveal that the oxidation of the complexes is partially reversible and to make the quantitative evaluation of the reversibility. The degree of reversibility of complex **2** corresponds to 0.7, while this value is equal to 0.24 for complex **1**. Both complexes on the semi-differential curves have similar E_semidiff_ values (equivalent to E_1/2_), but there are differences in E_onset_ potentials of about 55 mV. Thus, it is worth assuming that complex **1** exhibits greater solvent-induced transformations under the oxidation in DMF solutions than complex **2**.

The CV and semi-differential CV curves recorded for complexes in the solid state (Fig. 5b,d) reveal the single oxidation peaks (0.66 V for **1** and 0.61 V for **2**), and reversibility extents close to 1.0. The greater oxidation reversibility of complexes **1** and **2** in the solid state than that in the solutions argues for their greater chemical stability in the solid *vs* dissolved states. This allows to hypothesize that the complexes incorporated into the colloidal phase can demonstrate greater chemical stability toward the oxidation.

### ROS generation by F-127-*1*(*2*) NPs

**Fig. 6:**
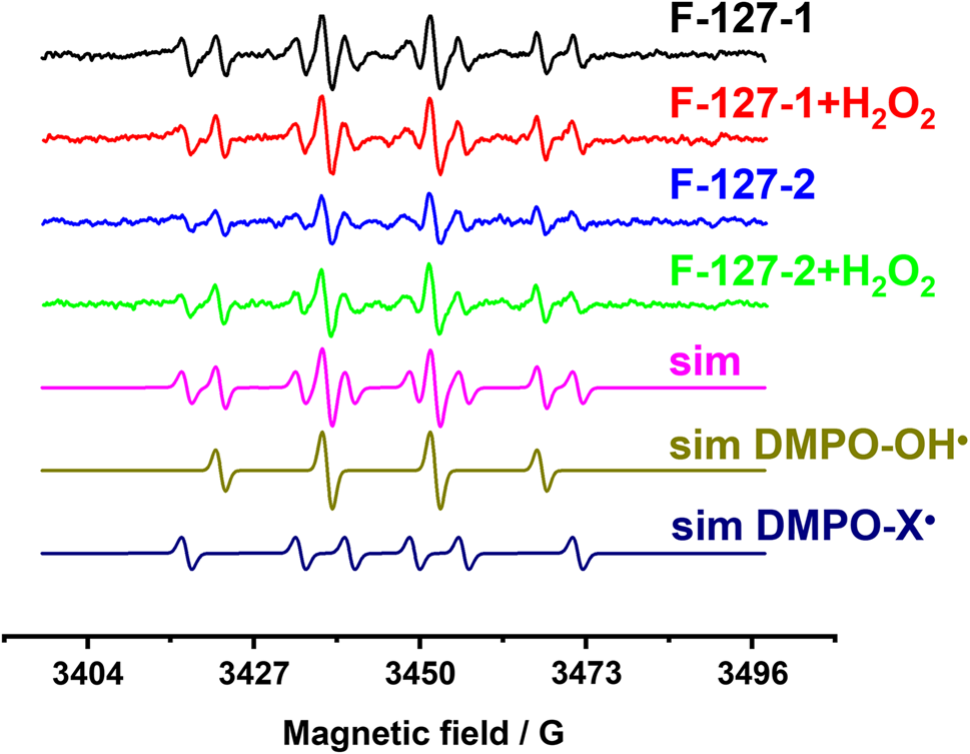
Experimental ESR spectra of F-127-**1**(**2**) NPs without and in the presence of H_2_O_2_, and simulated spectra of F-127–**1**(**2**) NPs (sim), DMPO-OH· and DMPO-R· spin-adducts

The spin trap-facilitated ESR technique is the well-known and widely used tool in detection of ROS. The detection derives from the well-defined signal of the long living adducts DMPO-OH· resulted from the interaction of the spin trap 5,5-Dimethyl-1-Pyrroline-N-Oxide (DMPO) with hydroxyl radicals (OH·) [39–41]. The well-defined signals of DMPO-OH· adducts characterized by g = 2.0055, a_N_ = 14.8 G, a_H_ = 14.8 G, ∆H = 0.7 G become detectable within 15 min after the addition of DMPO to F-127-**1**(**2**) NPs. The signals in both colloidal systems are contributed by DMPO-X· adducts (g = 2.0055, a_N_ = 15.8 G, a_H_ = 22.6 G, ∆H = 0.8 G), where X is organic radicals (Fig. 6). The intensity of DMPO-OH· signal is higher in the aqueous colloids of F-127-**1** vs. F-127-**2**, and the signal intensity remains unchanged under the addition of H_2_O_2_, which is quite different from F-127-**2**, where the signal intensity increases in the presence of H_2_O_2_.

The ROS generation via direct one electron reduction of oxygen by Cu(I)-based reductants is not thermodynamically favored, which argues for more strong oxidants as triggers for the Fenton-like transformations [13, 27]. The presence of hydrogen peroxide is commonly demonstrated as the main prerequisite for catalytic activity of Cu(I) complexes in Fenton-like reactions due to Equilibrium 1 [1, 3–8].

1 $${\text{Cu}}^{ + } + {\text{ H}}_{{2}} {\text{O}}_{{2}} + {\text{ H}}^{ + } = {\text{ Cu}}^{{{2} + }} + {\text{ OH}}\cdot \, + {\text{ H}}_{{2}} {\text{O}}$$

Both literature and the represented results demonstrate the self-boosting ROS generation by Cu(I)-based NPs in neutral conditions and without hydrogen peroxide [9]. This argues for the self-generated strong oxidants, such as H_2_O_2_ as the trigger of the Fenton-like reactions. However, only for F-127-**2** NPs the level of DMPO-OH· adducts is enhanced in the presence of H_2_O_2_, while F-127-**1** generate ROS independently on H_2_O_2_ (Fig. 6). It is worth noting that the aforesaid tendency correlates with the difference in the luminescent responses of F-127–**1**(**2**) to H_2_O_2_ (Fig. 4), but disagrees with the efficient self-maintained generation of DMPO-OH· by F-127–**1**. One can hypothesize the reductive potential of Cu(I) complexes as the reason for production of H_2_O_2_ via Equilibrium 2.

2 $${\text{2Cu}}^{ + } + {\text{ O}}_{{2}} + {\text{ 2H}}_{{2}} {\text{O }} = {\text{ 2Cu}}^{{{2} + }} + {\text{ H}}_{{2}} {\text{O}}_{{2}} + {\text{ 2OH}}^{-}$$

In turn, the production of H_2_O_2_ via the equilibrium (2) should be dependent on an accessibility of the Cu(I) centers to water and oxygen molecules. This, in turn, may be related to the chemical stability of the complexes incorporated into F-127-**1**(**2**). It is worth noting the lower chemical stability of complex **1** *vs* **2** revealed under the electrochemical oxidation of the complexes in the DMF solutions (Fig. 5). However, the represented results are insufficient to correlate the self-maintained ROS generation observed in the aqueous colloids with the chemical stability of the complexes revealed from the electrochemical measurements in the DMF solutions.

### Potential of F-127-*1*(*2*) NPs in cell therapy and labeling

The effect of the initial complexes **1**, **2** and F-127-**1**(**2**) NPs on the cell viability was measured for a number of cancer cell lines represented by M-HeLa, MCF-7 and HuTu 80, and normal cells (Chang Liver). The IC_50_ values calculated from the cell viability data (Fig. S16) are collected in Table 2. The comparative analysis of the IC_50_ values (Table 2) indicates that the cytotoxicity of complexes **1** and **2** is comparable with the literature data on molecular Cu(I) complexes [11–14, 27]. However, the IC_50_ values of the complex-based nanomaterials significantly differ from those of the complexes introduced in the form of the aqueous DMSO solutions. (More details are in the SI.) In particular, the IC_50_ values of F-127-**1**(**2**) are significantly higher than those of the complexes for M-HeLa, MCF-7 and Chang Liver cell lines, while the IC_50_ values of NPs are significantly lower for HuTu 80 compared to other cancer and the normal cell lines (Table 2). Moreover, the IC_50_ values of complexes **1** and **2**, assessed for cancer and normal cells, are quite close to each other, indicating insignificant anticancer specificity, quantified as the ratio of the IC_50_ values measured for cancer and normal cell lines. The anticancer specificity in the case of HuTu 80 cells increases on going from the complexes to the NPs. The different effects on the cell viability of complexes **1**, **2** introduced as the aqueous DMSO solutions and those incorporated into the NPs agree well with the different mechanisms contributing to the cytotoxicity of molecular and nanoparticulate forms of complexes [8, 10, 11, 13–15, 42, 43]. It is worth noting very poor if any luminescence of complexes **1**, **2** in the aqueous DMSO solutions, which argues for the degradation of the complexes in these conditions. Both aggregation of the complexes into nanomaterial and the hydrophilic coating of the latter significantly restrict the chemical transformations of the complexes in bio-environment, and thus, the IC_50_ values of the nanosystems based on Cu(I) complexes can be even above 100 μM [22].

**Table 2:** IC_50_ values determined for M-HeLa, MCF-7, HuTu 80 and Chang Liver cell lines after the incubation by complexes **1**, **2** and corresponding F-127-**1**(**2**) NPs

	IC_50_, µM			
	M-HeLa	MCF-7	HuTu 80	Ch Liver
1	25.9 ± 2.9	32.2 ± 2.3	23.5 ± 7.6	31.0 ± 0.2
2	29.9 ± 4.4	30.17 ± 0.01	35.2 ± 0.1	31.2 ± 4.3
F-127-**1**	> 50	> 50	29.3 ± 0.6	> 50
F-127-**2**	> 65	67.5 ± 1.0	38.9 ± 0.2	> 65

**Fig. 7:**
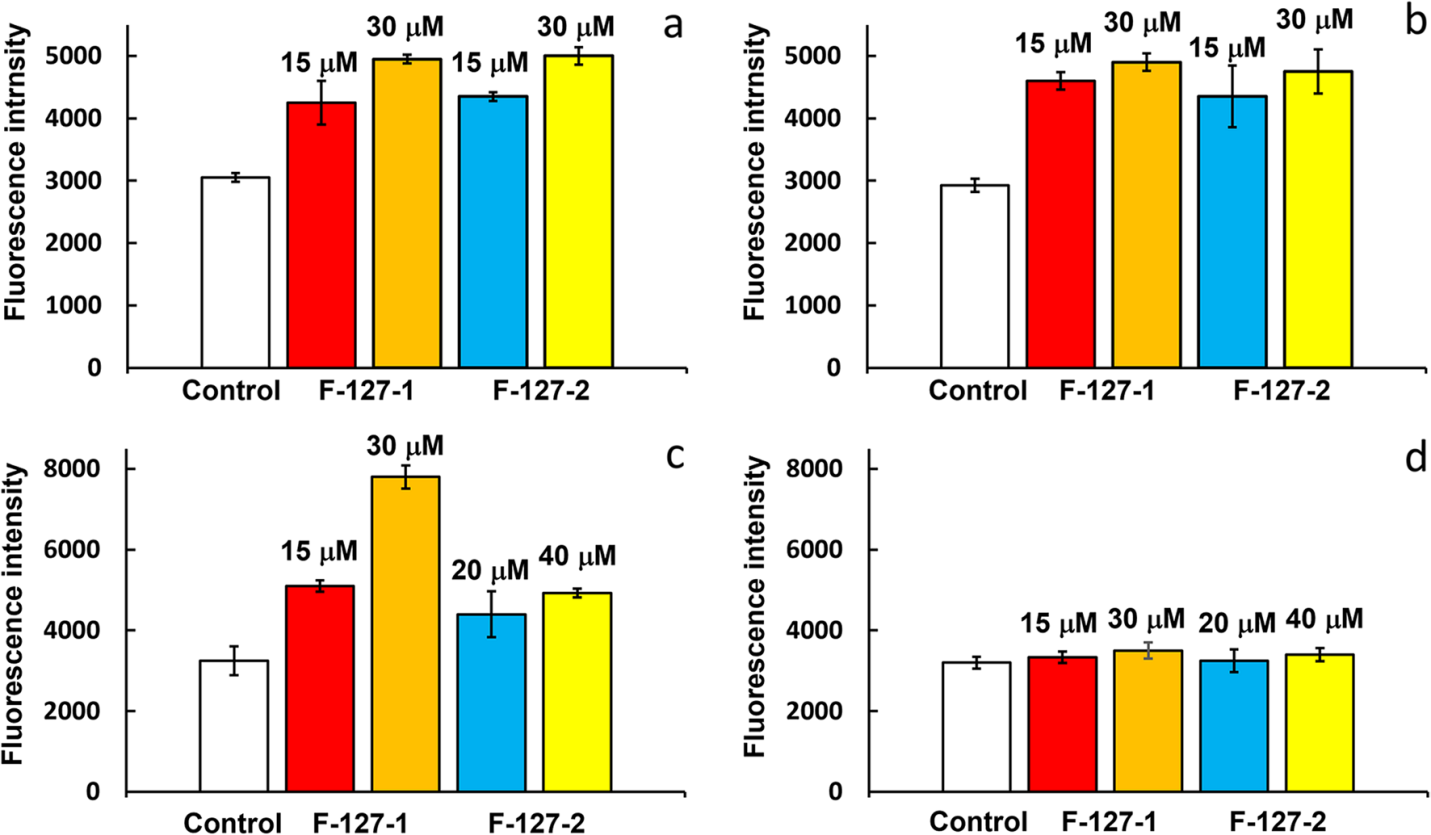
Study of cellular uptake (**a**, **b**) and intracellular ROS production (**c**, **d**) of F-127-**1** and F-127-**2** NPs on HuTu 80 (**a**, **c**) and M-HeLa (**b**, **d**) cell lines at different concentrations

The comparison of the IC_50_ values of F-127-**1**(**2**) for M-HeLa and MCF-7 cells with the values for Chang Liver cells reveals no anticancer specificity (Table 2). The cancer cell specificity of F-127-**1**(**2**) NPs revealed for HuTu 80 can derive from many factors, including a cell-dependent cellular uptake of the NPs. The luminescence of F-127-**1**(**2**) NPs allows to reveal their cell internalization, which was demonstrated by fluorescence microscopy (Figs. S17, S18) and quantitatively evaluated by flow cytometry (Fig. 7a, b) techniques for HuTu 80 and M-HeLa cell lines. The flow cytometry results indicate no significant differences between the cell lines for both F-127-**1**(**2**) NPs.

The analysis of the intracellular ROS production in HuTu 80 and M-HeLa cells incubated with F-127-**1**(**2**) both indicates the higher level of oxidative stress for HuTu 80 *vs* M-HeLa cells and reveals the greater ROS production in HuTu 80 cells by F-127-**1** vs. F-127-**2** (Fig. 7c,d). This agrees well with both IC_50_ values (Table 2) and in vitro ESR measurements (Fig. 6). Thus, the higher anticancer specificity (HuTu 80/Chang Liver) of F-127-**1** compared to F-127-**2** correlates with the levels of ROS generated in aqueous colloids. At the same time, the cytotoxicity of F-127-**1**(**2**) NPs does not correlate with the ability of F-127-**2** to generate greater level of ROS after the addition of hydrogen peroxide (Fig. 6). This contradicts with the statement that the intracellular level of H_2_O_2_ is of great impact on the intracellular ROS generation [22–25].

**Fig. 8:**
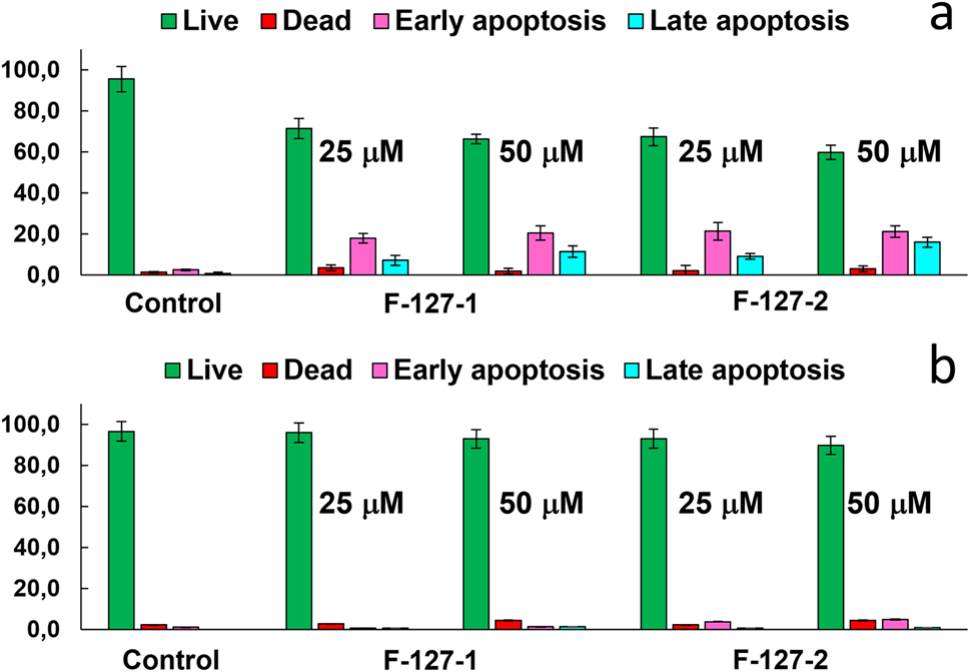
Flow cytometry analysis of HuTu 80 (**a**) and M-HeLa (**b**) cells treated with different concentrations of F-127-**1** and F-127-**2** NPs after Annexin V and PI staining. The values are presented as the mean ± SD (n = 3)

The oxidative stress induced by the ROS generation is commonly followed by the apoptotic pathway of the cells’ death. The apoptotic assay reveals the concentration dependent appearance of both early and late apoptotic cells in the sample of HuTu 80 cells incubated with F-127-**1**(**2**) NPs, while the insignificant extent of such cells is revealed for M-HeLa cell line incubated with the same amounts of F-127-**1**(**2**) (Figs. 8 and S19).

The high cytotoxicity and insignificant anticancer specificity of complexes **1** and **2** (Table 2) are associated with the non-apoptotic cell death pathways (Fig. S20), while the latter insignificantly contribute to the cytotoxicity of F-127-**1**(**2**) NPs.

**Fig. 9:**
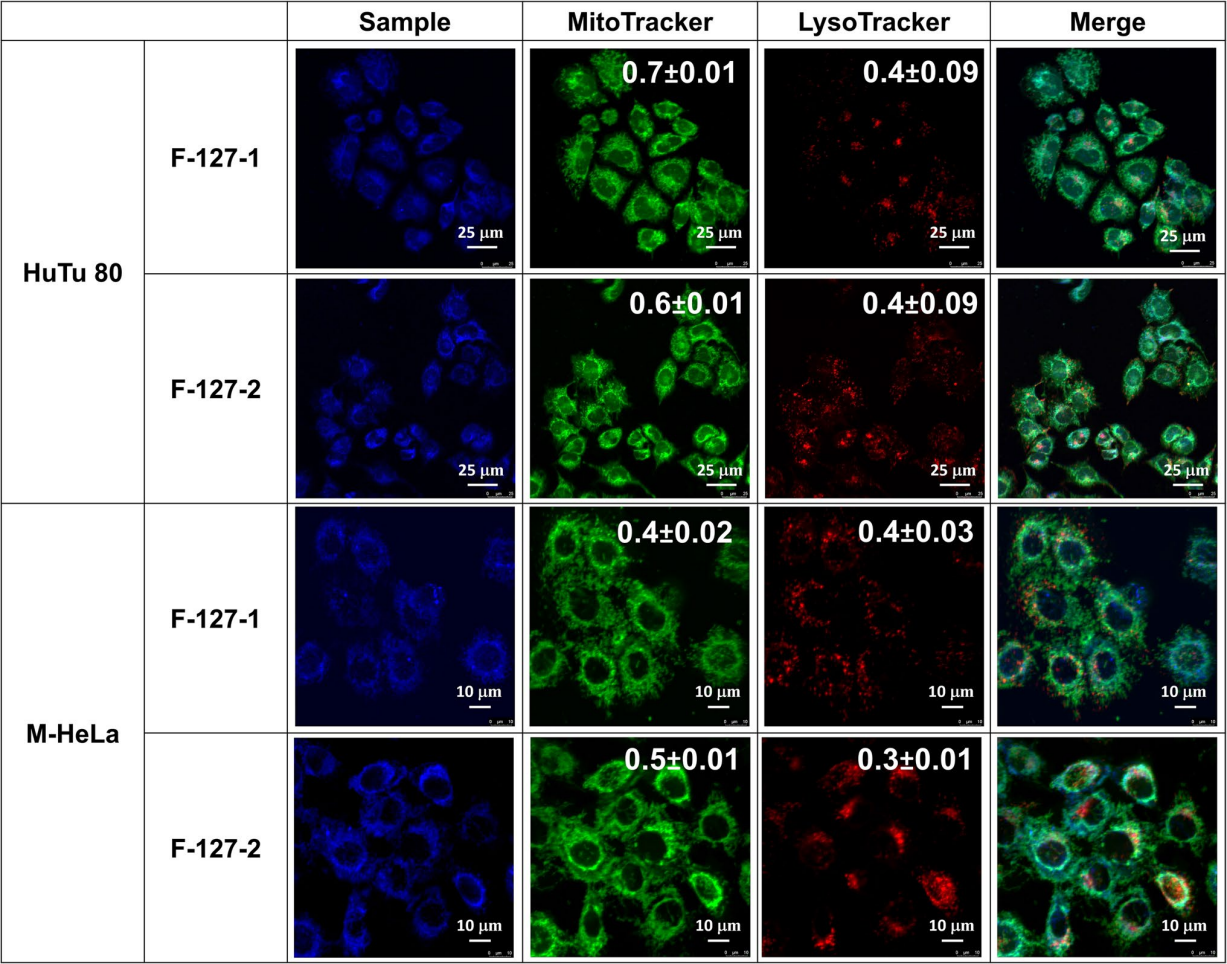
Colocalization analysis of F-127–**1**(**2**) NPs in different cellular compartments of HuTu 80 and M-HeLa cells after 24 h of incubation. The PCC values for mitochondrial and lysosomal compartments are presented in the corresponding cell images

The detailed analysis of the intracellular trafficking was performed for M-HeLa and HuTu 80 cell lines in order the reveal the reason for the observed cancer cell specificity of F-127-**1**(**2**) NPs. The confocal microscopy imaging of HuTu 80 and M-HeLa cells incubated with F-127-**1**(**2**) and co-incubated with MitoTracker and LysoTracker was performed (Fig. 9). The colocalization extents of the dyes and the NPs in the mitochondrial and lysosomal compartments are quantitatively presented through the Pearson correlation coefficients (PCC) values in Fig. 9.

In accordance with the PCC values, the colocalization extent in lysosomal compartments is low for both cell lines (Fig. 9), since PCC < 0.5 is considered as evidence of low colocalization extent [44]. However, localization in mitochondrial compartments is dependent on the cell nature. In particular, the PCC values evaluated for HuTu 80 cells are 0.7 ± 0.01 and 0.6 ± 0.01 for F-127-**1** and F-127-**2** correspondingly, while these values do not exceed 0.5 in M-HeLa cells.

**Fig. 10:**
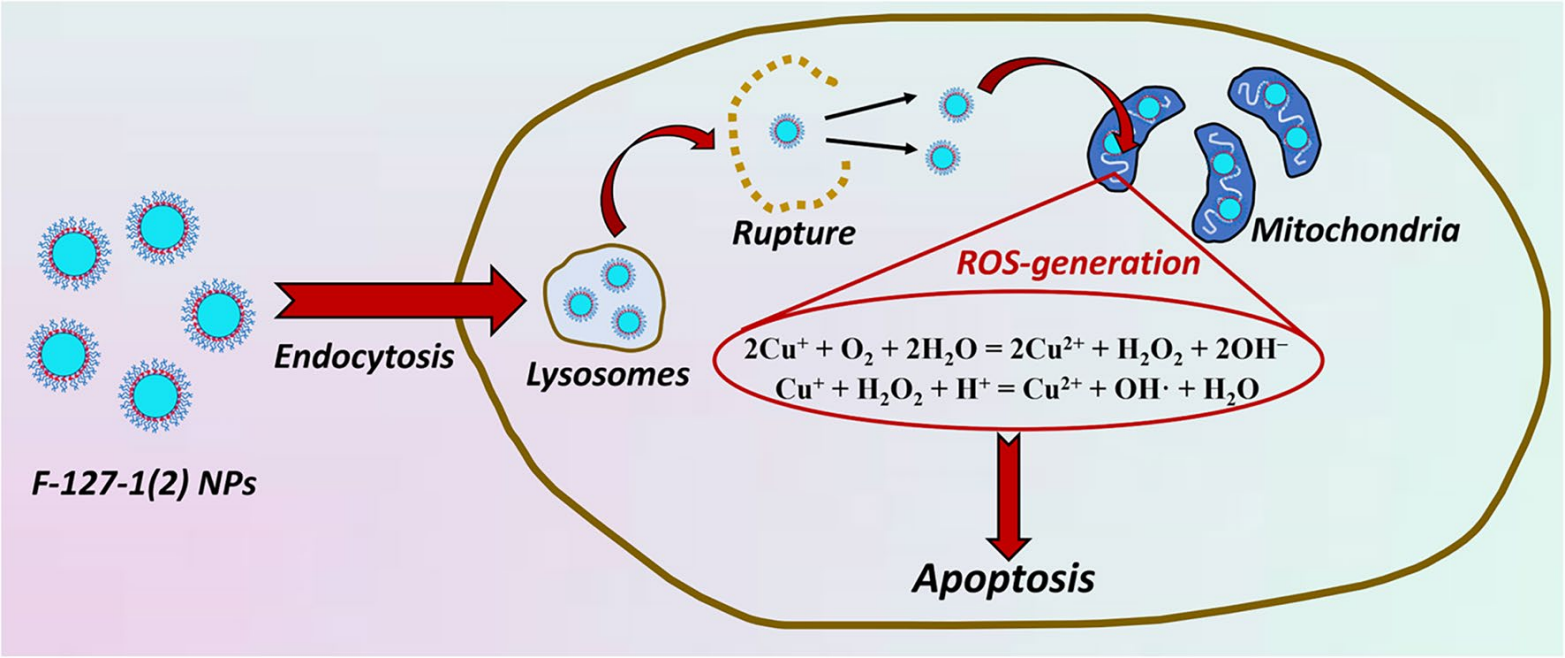
Cartoon illustration of the intracellular trafficking and CDT effect of F-127-**1**(**2**)

The great impact of mitochondria in such relevant biochemical processes as cell signal transduction, redox balance, biotransformation of amino acids and lipids, calcium homeostasis, apoptosis and programmed cell death makes mitochondrial compartments a focus of current interest in treating of cancer and cardiovascular diseases [45, 46]. Thus, the greater entering of the NPs into mitochondrial compartments can be associated with the greater intracellular oxidative stress, in turn, resulting in the apoptotic pathway of the cell death (Figs. 7 and 8), which is schematically represented by the cartoon figure (Fig. 10). However, this is not the only factor determining the cancer cell specificity, since commonly cancer cells exhibit an enhanced resistance to apoptosis, and the latter is influenced by a cancer genetics [47–49].

## Conclusion

The present work represents structure optimization of macrocyclic P_2_N_2_-ligands for developing the Cu(I) complexes exhibiting both efficient luminescence and enough chemical stability allowing the safe conversion of the complexes into luminescent hydrophilic NPs. The high colloidal stability of the developed NPs derived from their non-covalent surface modification makes them efficient cell markers. The cytotoxicity of the NPs is significantly lower than that of the corresponding molecular complexes, which agrees well with the stability of the NPs to chemical transformations in the solutions. The insignificant anticancer specificity of the complexes introduced in the form of the aqueous DMSO solutions is consistent with the non-apoptotic cell death pathways revealed for the cells incubated with the complexes. The cancer cell specificity of the NPs toward HuTu 80 cells mainly derives from the apoptotic cell death pathway, which correlates with the mitochondrial localization of the NPs and intracellular level of generated ROS.

### Supplementary Information

Supplementary information. The details of the Materials and Methods section, luminescence and IR spectra, DLS, PXRD and ICP-OES measurements results, flow cytometry data and fluorescence microscopy images can be found in Supplementary Material.

## Data Availability

Crystallographic data for the structures reported in this article have been deposited at the Cambridge Crystallographic Data Centre, under deposition numbers CCDC 2270682 (complex **1**), 2270680 (complex **3**). Copies of the data can be obtained free of charge via https://www.ccdc.cam.ac.uk/structures/. All other relevant data generated and analyzed during this study are included in this article and its supplementary information.

## Code availability

Source code is available upon request from the corresponding author.
