# Supplementary material for: ROS-producing nanomaterial engineered from Cu(I) complexes with P2N2-ligands for cancer cells treating
Source: Discov Nano. 2023 Oct 30;18(1):133. doi: 10.1186/s11671-023-03912-7 (PMC10616039; doi:10.1186/s11671-023-03912-7)
Supplement: Supplementary file 1 — Supplementary information. The details of the Materials and Methods section, luminescence and IR spectra, DLS, PXRD and ICP-OES measurements results, flow cytometry data and fluorescence microscopy images can be found in Supplementary Material. [file 11671_2023_3912_MOESM1_ESM.docx]

**ROS-producing nanomaterial engineered from Cu(I) complexes with P_2_N_2_-ligands for cancer cells treating**

Bulat A. Faizullin^1,^ *, Irina R. Dayanova^1^, Alexey V. Kurenkov^1^, Aidar T. Gubaidullin^1^, Alina F. Saifina^1^, Irek R. Nizameev^2^, Kirill V. Kholin^3^, Mikhail N. Khrizanforov^1,4^, Aisylu R. Sirazieva^1^, Igor A. Litvinov^1^, Alexandra D. Voloshina^1^, Anna P. Lyubina^1^, Guzel V. Sibgatullina^5^, Dmitry V. Samigullin^5,6^, Elvira I. Musina^1^, Igor D. Strelnik^1,4^, Andrey A. Karasik^1^, Asiya R. Mustafina^1^

***^1^ Arbuzov Institute of Organic and Physical Chemistry, FRC Kazan Scientific Center of RAS****, 8 Arbuzov str., 420088, Kazan, Russia*

*^2^ Department of Physics, Kazan National Research Technological University, 68 Karl Marx str., 420015, Kazan, Russia*

*^3^ Department of Nanotechnology in Electronics, Kazan National Research Technical University Named after A.N. Tupolev-KAI, 10 K. Marx Street, 420111 Kazan, Russia*

*^4^ Aleksander Butlerov Institute of Chemistry, Kazan Federal University, 1/29 Lobachevski str., 420008, Kazan, Russia*

*^5^ Kazan Institute of Biochemistry and Biophysics, FRC Kazan Scientific Center of RAS, 2/31 Lobachevski str., 420111, Kazan, Russia*

*^6^ Institute for Radio-Electronics and Telecommunications, Kazan National Research Technical University Named after A.N. Tupolev-KAI, 10 K. Marx Street, 420111 Kazan, Russia*

**Corresponding author*

E-mail address: [bulat_fayzullin95@mail.ru](mailto:bulat_fayzullin95@mail.ru)

ORCID: 0000-0003-4295-0891

Materials and Methods

Reagents and Materials

Hydrogen peroxide (H_2_O_2_, 30%), glutathione (GSH), 5,5-Dimethyl-1-Pyrroline-N-Oxide (DMPO) and Pluronic F-127 were purchased from Sigma-Aldrich. Acetic-acetate and phosphate were used as the buffers.

All reactions and purification manipulations were carried out under a dry argon atmosphere by using standard vacuum-line techniques. Commercially available solvents were purified, dried, deoxygenated and distilled before use [1]. Primary phosphine PyCH_2_CH_2_PH_2_ was obtained using the standard method described by Redmore [2]. Ligands **L3** and **L4** were obtained according previously described methods [3, 4] by the condensation reaction of PyCH_2_CH_2_PH_2_, paraform and primary amines (p-toluidine or benzhydrylamine).

**4-Iodophenyl-O,O-diethylphosphonate.** The mixture of 1,4-diiodobenzene (14.59 g, 44.2 mmol) and anhydrous NiCl_2_ (0.502 g, 10 mol%) was heated at 160 ^o^C. During the heating the P(OEt)_3_ (7.35 g, 44.2 mmol) was added dropwise via syringe. The byproduct EtI was distilled permanently during the reaction. The reaction mixture was kept under heating for 4 h. The products of the reaction have been separated via the chromatography. Hexane was used to remove unreacted diiodobenzene, the product was eluted by dichloromethane. Evaporation of dichloromethane led to the yellowish oily pure product. Yield: 7.35 g (65 %). ^1^H NMR (400 MHz, CDCl_3_): δ_H_ 7.80 (dd, ^4^*J*_PH_ = 3.3, ^3^*J*_HH_ = 8.2 Hz, 2H; H-C_6_H_4_), 7.49 (dd, ^3^*J*_HH_ = 8.2 Hz, ^3^*J*_PH_ = 13.2 Hz, 2H; H-C_6_H_4_), 4.01-4.15 (m, 4H; H-OCH_2_CH_3_), 1.28 (tr., ^3^*J*_HH_ = 7.1 Hz, 6H; H-OCH_2_CH_3_). ^31^P{^1^H} NMR (162 MHz, CDCl_3_): δ_P_ 18.05 ppm.

**4-Biphenyl-O,O-diethylphosphonate.** To the mixture of 4-Iodophenyl-O,O-diethylphosphonate (8.99 g, 26.5 mmol) and phenylboronic acid (3.54 g, 29.4 mmol) in ethanol (Ph_3_P)_4_Pd (0.92 g, 0.8 mmol) and 60 ml of 2M aqueous solution of Na_2_CO_3_ (14.0 g) was added. The reaction mixture was heated at 80 ^o^C during 10 h. After that 50 ml of water was added and water layer extracted with 50 ml of dichloromethane 3 times. Organic layer was combined and dried over MgSO_4_. The solvent was removed under vacuum and mixture was purified by the column chromatography. The product was eluted with ethyl acetate. The removing of the solvent under vacuum led to the white crystalline pure product. Yield: 7.5 g (98 %). ^1^H NMR (400 MHz, CDCl_3_): δ_H_ 7.85 (dd, ^3^*J*_HH_ = 8.0, ^3^*J*_PH_ = 13.1 Hz, 2H; H-C_6_H_4_), 7.65 (dd, ^3^*J*_HH_ = 8.0 Hz, ^4^*J*_PH_ = 3.9 Hz, 2H; H- C_6_H_4_), 7.57 (d, ^3^*J*_HH_ = 7.4 Hz, 2H; H- C_6_H_5_), 7.42 (dd, ^3^*J*_HH_ = 7.8 Hz, ^3^*J*_HH_ = 7.4 Hz, 2H; H- C_6_H_5_), 7.35 (d, ^3^*J*_HH_ = 7.8 Hz, 1H; H-C_6_H_5_), 4.03-4.22 (m, 4H; H-OCH_2_CH_3_), 1.32 (tr., ^3^*J*_HH_ = 7.1 Hz, 6H; H-OCH_2_CH_3_). ^31^P{^1^H} NMR (162 MHz, CDCl_3_): δ_P_ 18.97 ppm.

**4-Biphenylphosphine.** To suspension of LiAlH_4_ (1.42 g, 37.4 mmol) in 50 ml of diethyl ether a solution of 4-biphenylphosphonate (7.77 g, 26.8 mmol) in 50 ml of diethyl ether was added dropwise at -2 - 0 ^o^C. The reaction mixture kept stirring at room temperature for 20 h. After that degassed water (40 ml) was added dropwise at -2 - 0 ^o^C to decompose the excess of LiAlH_4_. The organic layer was separated to the flask with MgSO_4_, the aqueous layers was washed by diethyl ether 3 times. All organic layers were combined and dried over MgSO_4_ for 12 h. After that the diethyl ether was removed by the distillation, the residue was distilled at 160 ^o^C under 5×10^-2^ mbar to give pure 4-biphenylphosphine. Yield: 2g (40 %). ^1^H NMR (400 MHz, CDCl_3_): δ_H_ 7.55-7.60 (m, 4H; H-C_6_H_4_), 7.52 (m, ^3^*J*_HH_ = 8.3 Hz, 2H; H- C_6_H_5_), 7.44 (m, ^3^*J*_HH_ = 8.3 Hz, ^3^*J*_HH_ = 7.4 Hz, 2H; H- C_6_H_5_), 7.35 (m, ^3^*J*_HH_ = 7.4 Hz, 1H; H- C_6_H_5_), 4.04 (d, ^1^*J*_PH_ = 203.9 Hz, 2H; H-PH_2_). ^31^P{^1^H} NMR (162 MHz, CDCl_3_): δ_P_ -123.7 (^2^*J*_PH_ = 203 Hz) ppm.

**1,5-bis(p-tolyl)-3,7-bis(biphenyl)-1,5-diaza-3,7-diphosphacyclooctane L1**. The mixture of biphenylphosphine (688 mg, 3.7 mmol) and paraform (222 mg, 7.4 mmol) was heated at 120 ^o^C until homogenization. Obtained oily adduct was dissolved in ethanol (5 ml) and to this solution *para*-toluidine (396 mg, 3.7 mmol) in 5 ml of ethanol was added. The reaction mixture was stirred at 80 ^o^C for 5 h to give white precipitate. After that the precipitate was filtered, washed with ethanol and recrystallized from toluene to give pure compound **L1**. Yield: 45 %. Elemental analysis calcd (%) for C_42_H_40_N_2_P_2_: C 79.49, H 6.31, N 4.42, P 9.78; found: C 79.26, H 6.43, N 4.21, P 9.53. ^1^H NMR (400 MHz, DMF-d7): δ_H_ 7.73-7.87 (m, 13H; H-C_6_H_4_ + H- C_6_H_5_), 7.54 (m, 4H; H- C_6_H_5_), 7.43 (m, 2H; H- C_6_H_5_), 7.02 (m, ^3^*J*_HH_ = 8.3 Hz, 4H; H-p-Tol), 6.76 (m, ^3^*J*_HH_ = 8.0 Hz, 4H; H-p-Tol), 4.66 (dd, ^3^*J*_HH_ = 14.2 Hz, ^2^*J*_PH_ = 5.4 Hz, 4H; H_a_-P-CH_2_-N), 4.35 (dd, ^3^*J*_HH_ = 14.2 Hz, ^2^*J*_PH_ = 25.6 Hz, 4H; H_b_-P-CH_2_-N), 2.17 (s, 6H; H-CH_3_(p-Tol)). ^31^P{^1^H} NMR (162 MHz, DMF-d7): δ_P_ -52.9 ppm.

**1,5-bis(benzhydryl)-3,7-bis(biphenyl)-1,5-diaza-3,7-diphosphacyclooctane L2** was obtained analogously to **L1** with the use of 4-biphenylphosphine (470 mg, 2.5 mmol), paraform (152 mg, 5.0 mmol) and benzhydrylamine (462 mg, 2.5 mmol). Yield: 47%. Elemental analysis calcd (%) for C_54_H_48_N_2_P_2_: C 82.44, H 6.11, N 3.56, P 7.89; found: C 82.28, H 6.18, N 3.45, P 7.80. ^1^H NMR (400 MHz, DMF-d7): δ_H_ 7.65 (d, ^3^*J*_HH_ = 7.3 Hz, 8H; H-Ph_2_CH), 7.48 (d, ^3^*J*_HH_ = 7.2 Hz, 4H; H-Ph_2_CH), 7.40 (dd, ^3^*J*_HH_ = 7.8 Hz, ^3^*J*_HH_ = 7.3 Hz, 4H; H- C_6_H_5_), 7.3 (m, 10H; H-Ph_2_CH + H- C_6_H_5_), 7.19 (m, 8H; H- C_6_H_5_+ H- C_6_H_4_), 6.53 (m, 4H; H- C_6_H_4_), 5.84 (br.s., 2H; H- Ph_2_CH), 3.71 (dd, ^3^*J*_HH_ = 14.3 Hz, ^3^*J*_HH_ = 14.8 Hz, 8H; H-P-CH_2_-N). ^31^P{^1^H} NMR (162 MHz, DMF-d7): δ_P_ -69.3 ppm.

**[Bis-κ^2^-(1,5-bis(p-tolyl)-3,7-bis(biphenyl)-1,5-diaza-3,7-diphosphacyclooctane)]copper(I) tetrafluoroborate 1.** To a solutions of tetrakis(acetonitrile)copper(I) tetrafluoroborate (Cu(CH_3_CN)_4_BF_4_ , 26.7 mg, 0.08 mmol) a solution of ligand **L1** (93 mg, 0.17 mmol) in DMF was added. The reaction mixture was stirred for 10 h at room temperature. After that the solvent was evaporated under vacuum to give white precipitate. The precipitate was washed by ethanol and diethyl ether 3 times and dried over vacuum. Yield: 95 mg (90%). Elemental analysis calcd (%) for C_84_H_80_CuN_4_P_4_: C 75.69, H 6.05, N 4.20, P 9.29, Cu 4.77; found: C 79.60, H 6.12, N 3.98, P 9.06, Cu 4.65. MS (ESI_pos_, m/z (I_rel_, %), ion): 1331 (100) [2L+Cu]^+^. ^1^H NMR (400 MHz, acetone-d6): δ_H_ 8.0 (m, 4H; H-BiPh), 7.67 (m, 4H; H-BiPh), 7.60 (m, 4H; H-BiPh), 7.54 (dd, ^3^*J*_HH_+^3^*J*_HH_ = 14.95 Hz, 4H; H-BiPh), 7.46 (m, 2H; H-BiPh), 7.14 (s, 8H; H-p-Tol), 4.52 (br.d, ^3^*J*_HH_ = 13.8 Hz, H_a_-P-CH_2_-N), 4.27 (br.d, ^3^*J*_HH_ = 13.8 Hz, H_b_-P-CH_2_-N), 2.26 (s, 6H; H-CH_3_(p-Tol)). ^31^P{^1^H} NMR (162 MHz, acetone-d6): δ_P_ -14.1 ppm.

**[Bis-κ^2^-(1,5-bis(benzhydryl)-3,7-bis(biphenyl)-1,5-diaza-3,7-diphosphacyclooctane)]copper(I) tetrafluoroborate 2** was obtained analogously to complex **1** with the use of ligand **L2** (152 mg, 0.194 mmol) and Cu(CH_3_CN)_4_BF_4_ (30.5 mg, 0.097 mmol). Yield: 137 mg (82%). Elemental analysis calcd (%) for C_108_H_96_CuN_4_P_4_: C 79.22, H 5.91, N 3.42, P 7.57, Cu 3.88; found: C 79.12, H 5.96, N 3.13, P 7.47, Cu 3.81. MS (ESI_pos_, m/z (I_rel_, %), ion): 1636 (100) [2L+Cu]^+^.^1^H NMR (400 MHz, CD_3_CN): δ_H_ 7.59 (d, ^3^*J*_HH_ = 8.0 Hz, 4H; H-Ph_2_CH or H-BiPh), 7.52 (d, ^3^*J*_HH_ = 7.3 Hz, 4H; H-Ph_2_CH or H-BiPh), 7.45 (dd, ^3^*J*_HH_ = 7.7 Hz, ^3^*J*_HH_ = 7.1 Hz, 4H; H-BiPh), 7.32-7.42 (m, 6H; H-BiPh), 7.26 (m, 8H; H-Ph_2_CH), 7.2 (m, 12H; H-Ph_2_CH+H-BiPh), 4.99 (br.s., 2H; H- Ph_2_CH), 3.57 (br.d, ^3^*J*_HH_ = 13.1 Hz, 4H; H_a_-P-CH_2_-N), 3.23 (br.d, ^3^*J*_HH_ = 13.1 Hz, 4H; H_b_-P-CH_2_-N). ^31^P{^1^H} NMR (162 MHz, CD_3_CN): δ_P_ -20.0 ppm.

**[Bis-κ^2^-(1,5-bis(p-tolyl)-3,7-bis(2-(pyridine-2‘-yl)ethyl)-1,5-diaza-3,7-diphosphacyclooctane)]copper(I) tetrafluoroborate 3.** To solution of ligand **L3** (0.25 g, 0.46 mmol) in acetonitrile (5 ml) a solution of Cu(CH_3_CN)_4_BF_4_  (0.07 g, 0.23 mmol) in acetonitrile (5 ml) was added. The reaction mixture was stirred for 24 h at room temperature. After that the solvent was evaporated under vacuum and white precipitate was formed. The precipitated was washed by diethyl ether and dried over vacuum. Yield: 0.23 g (82%). Mp 133 °C. MS (ESI_pos_, m/z (I_rel_, %), ion): 1143 (100) [2L+Cu]^+^. ^1^H NMR (CD_3_CN, ppm): δ_H_ 8.47 (dd., ^3^J_HH_=4.77, ^4^J_HH_=0.73 Hz, 2H, H- Py), 7.63 (ddd., ^3^J_HH_=7.70, ^3^J_HH_=5.87, ^4^J_HH_=1.83 Hz, 2H, H-Py), 7.17 (ddd., ^3^J_HH_=5.87, ^3^J_HH_=4.77, ^4^J_HH_=1.10 Hz, 2H, H-Py), 7.03 (d., ^3^J_HH_=7.70 Hz, 2H, H-Py), 7.00 (d., ^3^J_HH_=8.44 Hz, 4H, H- *p*-Tol), 6.88 (d., ^3^J_HH_=8.44 Hz, 4H, H- *p*-Tol), 3.73-3.84 (m., 8H, H- PCH_2_N), 2.91-2.98 (m., 4H, H- CH_2_CH_2_P), 2.20 (s., 6H, H- *p*-Tol), 2.20-2.13 (m., 4H, H- CH_2_CH_2_P). ^31^P{^1^H} NMR (CD_3_CN): δ_P_ -14.70 ppm.

**[Bis-κ^2^-(1,5-bis(benzhydryl)-3,7-bis(2-(pyridine-2‘-yl)ethyl)-1,5-diaza-3,7-diphosphacyclooctane)]copper(I) tetrafluoroborate 4** was obtained analogously to complex **3** with the use of ligand **L4** (0.1 g, 0.20 mmol) and Cu(CH_3_CN)_4_BF_4_ (0.03 g, 0.10 mmol). Yield 0.09 g (82%). MS (ESI_pos_, m/z (I_rel_, %), ion): 1447 (100) [2L+Cu]^+^. ^1^H NMR (CD_3_CN, ppm): δ_H_ 8.31 (d, ^3^J_HH_=3.94 Гц, 2H, H- Py), 7.49 (ddd, ^3^J_HH_=7.70, ^3^J_HH_=7.61, ^4^J_HH_=1.83 Гц, 2H, H-Py), 7.30-7.6 (m., 8H, H-Ph), 7.25-7.28 (m., 4H, H-Ph), 7.20-7.25 (m., 8H, H-Ph), 7.05-7.10 (m., 2H, H- Py), 6.80 (d, ^3^J_HH_=7.79, 2H, H- Py), 4.87 (s, 2H, H- CH(Ph)_2_), 3.06-3.14 (m., 4H, H- PCH_2_N), 2.86-2.93 (m., 4H, H- PCH_2_N), 2.74-2.83 (m., 4H, CH_2_CH_2_P), 1.99-2.06 (m., 4H, H- CH_2_CH_2_P). ^31^P{^1^H} ЯМР (CD_3_CN): δ_P_ -17.07 ppm.

**The aqueous F-127-1(2) colloids** were synthesized according to previously published procedure [5]. In particular, NPs were obtained by the drop-wise addition of 0.6 mL of the complex **1**(**2**) solution in DMF (C = 1 mM) to 2.4 mL of aqueous NaCl solution (C = 0.5 M) under vigorous stirring. The turbid solutions were then subjected to ultrasonic treatment within 20 minutes at room temperature with subsequent separation of colloids through centrifugation (15000 rpm for 35 minutes at 20 ^0^C). Then 3 mL of F-127 aqueous solution (C = 1 g/L) was added to the precipitate and also subjected to ultrasonic treatment followed by centrifugation. The ultrasonication-centrifugation procedure was repeated twice in order to remove the excess of F-127. Concentrations of the resulted aqueous colloids (0.1 mM and 0.13 mM for F-127-**1** and F-127-**2**, respectively) are given in terms of copper ion content determined by the ICP-OES. The loading efficiencies of complexes **1** (50%) and **2** (65%) in nanoparticles also calculated from ICP-OES results.

Methods

Elemental analysis

Elemental analysis was performed on a EuroVector-3000. The determination of the P and Cu content was provided by combustion in an oxygen stream.

NMR spectroscopy

The ^1^H NMR (400.13 MHz) and ^31^P NMR (161.96 MHz) spectra were recorded on a Bruker Avance 400 spectrometer by using the residual solvent as an internal reference for ^1^H (δ = 7.26 in CDCl_3_, δ = 5.31 ppm in CD_2_Cl_2_, δ = 1.94 ppm in CD_3_CN, δ = 8.03, 2.92, 2.75 ppm in DMF-d_7_, δ = 2.05 ppm in acetone-d_6_) and 85% aqueous solution of H_3_PO_4_ as an external reference for ^31^P. Chemical shifts are reported in ppm and coupling constants (J) are reported in Hz.

Dynamic light scattering

Nanoparticles’ diameters and zeta potentials were determined by dynamic light scattering on a Zetasizer Nano instrument (Malvern Instruments, U.K.). Zeta potentials were calculated by the Smoluchowski-Helmholtz equation [6]. The temperature during measurements were controlled at 25 °C. All measurements were repeated at least three times.

Fluorescence spectroscopy

Luminescence of nanoparticles was recorded on a fluorescence spectrophotometer Hitachi F-7100 (Tokyo, Japan). The samples were excited by 330 nm, 340 nm or 370 nm and the emission spectra were recorded in 350–600 nm region. Quantum yields were obtained on Fluorolog QM-75-22-C (Horiba) spectrofluorometer using integration sphere Quanta Phi (Horiba).

FTIR spectroscopy

The IR spectra were obtained on a Bruker Tensor 27 Fourier-transform spectrometer (Ettlingen, Germany) in 4000–400 cm^−1^ range with 4 cm^–1^ optical resolution. Accumulation of 32 scans of KBr pressed pellets (for complexes and NPs) and CaF_2_ windows (for liquid DMF) was performed.

ICP-OES

P and Cu ions content were identified by means of inductively coupled plasma optical emission spectrometer (ICP-OES) iCAP 6300 DUO by Varian Thermo Scientific Company equipped with a CID detector (168 Third Avenue, Waltham, MA 02451, USA). The 178.284 and 324.754 nm spectral lines were used to determine the content of P and Cu ions, respectively. 10 ppm of Sc standard in each sample was used as the internal standard. P and Cu standards were used for five-point calibration.

ESI measurements

ESI-mass spectra were recorded on AmaZon X ion trap mass spectrometer (Bruker Daltonics, Germany) in positive and negative modes for acetonitrile and DMF solutions of complexes.

TEM measurements

Samples were prepared as follows: a drop of 6 μL was taken from the middle of a freshly prepared solution using a dispenser (Biohit Proline Plus, Göttingen, Germany) and applied to a 300 mesh copper grid with a carbon-formvar support film (Agar Scientific, Essex, CM24 8GF, UK). A drop completely covers the grid. The sample preparation process is carried out at room temperature. Next, the sample is dried in a muffle furnace at 80 °C. TEM images were obtained by use of Hitachi HT7700 transmission electron microscope (Japan) at an accelerating voltage of 100 kV (direct observation state) [7].

Electrochemistry

Cyclic voltammetry (CV) for 0.5 mM Cu(I) complexes was carried out on an electrochemical analyser (BASi, EClipse). Three-electrode glass cell with a working electrode – glassy carbon (with diameter of 3.0 mm), a counter electrode – Pt wire, and a reference electrode – Ag wire (Ag/Ag^+^, 0.01 M AgNO_3_). All measurements were performed in dimethylformamide (DMF) with 0.1 M Bu_4_NBF_4_ (TBATFB) as a supporting electrolyte at room temperature [8].

For solid state measurement an RTIL-CPE [8] as working electrode (ð = 3 mm) was used with an Ag/AgCl (3 M NaCl) electrode as the reference electrode and a Pt wire as counter electrode. A 0.1 M Bu_4_NBF_4_ was used as supporting electrolyte for the measurement of current-voltage curves. The reference electrode was connected with the cell solution by a modified Luggin capillary filled with the supporting electrolyte solution (0.1 M Bu_4_NBF_4_ in CH_3_CN). Thus, the reference electrode assembly had two compartments, each terminated with an ultra-fine glass frit to separate the AgCl from the analyte [8].

Powder X-ray diffraction (PXRD)

PXRD measurements were performed on an automatic Bruker D8 Advance diffractometer equipped with a Vario attachment and Vantec linear PSD using Cu radiation (40 kV, 40 mA) monochromated by a curved Johansson monochromator (λ Cu Kα1 1.54063 Å) (Bruker Optik GmbH, Ettlingen, Germany). Room-temperature data were collected in the reflection mode with a flat-plate sample [9].

Dispersion of nanoparticles, which were synthesized without F-127 layer, was applied to a silicon plate. To increase the total amount of the sample, several more layers were applied on top of the first one after it dried. Patterns were recorded in the 2θ range between 3° and 80° in 0.008° steps with a step time of 0.1-4 s. The samples were spun (15 rpm) throughout the data collection. Processing of the obtained data performed using EVA [10] software package.

X-Ray Crystallography

Crystals were placed in glass capillary in random orientation. Data of crystal **3** were collected on a Bruker Kappa Apex II CCD diffractometer using graphite monochromated MoKα(λ = 0.71073 Å) radiation and φ and ω-scan rotation at temperature 100(2) K. Data of crystal **4** were collected on a Bruker D8 Quest CCD diffractometer using graphite monochromated MoKα (λ = 0.71073 Å) radiation and φ and ω-scan rotation at 150(2) K**.**

Data collection was indexed, integrated, and scaled using the APEX2 data reduction package and corrected for absorption using SADABS [11, 12]. The structures were solved by direct methods using SHELXT program and refined using SHELXL program [13, 14].

**Crystal Data, Structure Refinement for complex 1:** C_84_H_80_CuN_4_P_4_, BF_4_ [+ solvent], *M* = 1419.76, tetragonal, space group P4/nnc, Z = 4 (complex in a special position on inversion axe -4, anion BF_4_ disordered by 222 axes). At 150 K *a* = *b* = 20.1943(12), *c* = 26.1957(15), Å, *V* = 10682.9(14) Å^3^, *D*calc = 0.883 g cm^–3^; *μ*(MoKα) = 0.304 mm^–1^; ΘMax = 28.3°, 254083 reflections measured, 6643 independent reflections, 2470 reflections with *I* ≥ 2σ(*I*). All non-hydrogen atoms of structure were refined anisotropically, H atoms were calculated on idealized positions and refined as riding atoms. Structure weas refined using programs of WinGX Program package [15].

When the structure was refined, in the difference Fourier electron density maps intense peaks were solved but attempts to identify solvate molecules from them were unsuccessful. When refining solvate molecules, unrealistic geometric parameters and parameters of anisotropic atomic displacements were obtained. Solvate molecules are strongly disordered by the symmetry elements of the crystal. As a result, it was decided to refine the structure with an undetermined solvent using the SQEEZE procedure of the PLATON program [16]. It was calculated that in the unit cell of the crystal 4180 Å^3^ (40% of the cell volume) is filled with undefined solvent molecules, which can contain 1625 electrons. Thus, the calculated density and linear absorption coefficient of the crystal do not correspond to the real ones. Final *R*1 = 0.0983, *R*w = 0.2631 for observed reflections, and *R*1 = 0.2218, *R*w = 0.3377 for all reflections; goodness of fit 0.964.

**Crystal Data, Structure Refinement for complex 3:** 2(C_64_H_76_CuN_8_P_4_), 2(BF_4_), CH_2_Cl_2_, 2(H_2_O), Sum Formula C_129_H_158_B_2_Cl_2_Cu_2_F_8_N_16_O_2_P_8_; *M* = 2584.10, triclinic, space group *P-1,* Z =1. Crystal of the compound is hydrate and hemisolvate with methylenechloride, solvate molecule is disordered around the center of symmetry. At 100 K *a* = 12.4493(8), *b* = 16.2536(10), *c* = 16.5673(12) Å, α = 102.964(4), β = 92.968(4), γ = 104.679(3), *V* = 3139.2(4) Å^3^, *D*calc = 1.367 g cm–3; *μ*(MoKα) = 0.554 mm^–1^; ΘMax = 27.0°, 60116 reflections measured, 13680 independent reflections, 9160 reflections with *I* ≥ 2σ(*I*). All non-hydrogen atoms of structure were refined anisotropically, H atoms were calculated on idealized positions and refined as riding atoms. Structure was refined using programs of Olex2 1.2 Program package [17]. Final *R*1 = 0.0568, *R*w = 0.1467 for reflections with *I* ≥ 2σ(*I*), and *R*1 = 0.1002, *R*w = 0.1961 for all reflections, goodness of fit 1.068.

Deposition numbers CCDC 2270682 (complex **1**) and 2270680 (complex **3**) contain the supplementary crystallographic data for this paper. These data are provided free of charge by the joint Cambridge Crystallographic Data Centre and Fachinformationszentrum Karlsruhe Access Structures service [www.ccdc.cam.ac.uk/structures](http://www.ccdc.cam.ac.uk/structures).

Analysis of molecular and crystal structures were made with program PLATON [18], figures were prepared by program MERCURY [19].

ESR measurements

The ESR measurements were carried out on X-band ELEXSYS E500 ESR spectrometer. ESR spectra were simulated using a WinSim 0.96 program (developed by NIEHS). The samples were buffered (pH = 7.0) solutions of DMPO (C = 0.1 M) in the presence of nanoparticles (C = 10 μM (F-127-**1**) and 13 μM (F-127-**2**)) with/without H_2_O_2_ (C = 100 μM). Measurements were performed at 310 K [20].

Cytotoxicity assay

Cytotoxicity of the nanoparticles on human normal and cancer cells was determined by means of the MTT test [21]. The M-HeLa clone 11 human, epithelioid cervical carcinoma, strain of HeLa, clone of M-HeLa; human breast adenocarcinoma cells (MCF-7); human duodenal cancer cell line (HuTu 80) from the Type Culture Collection of the Institute of Cytology (Russian Academy of Sciences) and Chang liver cell line (Human liver cells) from N. F. Gamaleya Research Center of Epidemiology and Microbiology were used in experiments. After seeding on a 96-well plate (Nunc) at a concentration of 5×10^3^ cells per well in a volume of 100 μL of medium cells were cultured in a CO_2_ incubator at 37 °C until a monolayer was formed. Afterwards, the nutrient medium was removed and 100 µL of solutions of the test samples in given dilutions were added to the wells, the samples of the aqueous colloids were diluted by the nutrient medium, while the addition of 5% DMSO was performed under the introduction of the complexes itself. After 24 h of cell incubation with either colloids or solutions of the complexes, the nutrient medium was removed from the plates, with further addition of 100 μL of serum-free nutrient medium with MTT at a concentration of 0.5 mg/mL. Then, the cells were incubated for 4 h at 37 °C. In order to dissolve the intracellular crystals of formazan 100 µL of DMSO was added in each well. Optical density was recorded at 540 nm using an Invitrologic tablet reader (Russia, Novosibirsk). The experiments were performed in triplicate. The untreated cells were used as controls.

The IC_50_ values were calculated using the online calculator MLA - Quest Graph™ IC_50_ Calculator (AAT Bioquest, Inc, Sunnyvale, CA, USA) (Version 2021) (accessed on 12 May 2023) [22]. The values calculated from the triplicate measurements were averaged.

Cellular uptake study

M-HeLa cell lines (1×10^5^ cells/well) in a final volume of 500 µL were plated in 24-well plates (Eppendorf). After 24-hour incubation, the aqueous dispersions of the nanoparticles were added to the wells at concentrations of 15 μM and 30 μM and incubated for 24 hours under CO_2_. Cellular uptake was analyzed by flow cytometry (Guava easy Cyte 8HT, MERCK, Kenilworth, USA). Untreated cells were used as a negative control. The studies were carried out in triplicate. The values are presented as the mean ± SD (p < 0.05) [23].

Cell apoptosis analysis

M-HeLa cells (1×10^6^ cells per well) in a ﬁnal volume of 2 mL were sown in 6-well plate. After 24-hour incubation, the aqueous dispersions of the nanoparticles were added to the wells. The cells were harvested at 2000 rpm for 5 min and then washed twice with ice-cold PBS (4 ^o^C), followed by resuspension in 100 μL of the binding buffer. Next, the samples were incubated with 0.35 μL of Annexin V-Alexa Fluor 647 and 0.1 μL of PI for 40 min at room temperature in the dark [24]. Finally, the cells were analyzed by ﬂow cytometry (Guava easy Cyte, MERCK, Kenilworth, USA). Untreated cells were used as control. The 20000 events have been analyzed in the apoptotic assay. The studies were carried out in triplicate. The values are presented as the mean ± SD (p < 0.01).

Detection of intracellular ROS

M-HeLa cells were incubated with the aqueous dispersions of the nanoparticles at various concentrations for 24 h. ROS generation was investigated using flow cytometry assay and CellROX® Deep Red flow cytometry kit [25]. For this, M-HeLa cells were harvested at 2000 rpm for 5 min and then washed twice with ice-cold PBS (4 ^o^C). The separated cells were resuspended in 0.1 mL of medium without FBS, to which 0.2 μL of CellROX® Deep Red was added with the following incubation at 37 ^o^C for 30 min. After the washing of the cells for three times, they were suspended in PBS, and the production of ROS in the cells was immediately monitored using flow cytometer (Guava easy Cyte, MERCK, Kenilworth, USA). The experiments were performed in triplicate. Data are presented as mean ±SD (p<0.05).

Fluorescence microscopy

M-HeLa cells (1×10^5^ cells per well) in a final volume of 2 mL were seeded into 6-well coverslip plates, the cells were incubated for 24 hour. Then, the aqueous colloids at the concentrations designated in Figs S17, S18 were added to the wells with further culturing for 24 hours in a CO_2_ incubator. Then, the incubated M-HeLa cells were fixed and stained with Rhodamine B. Experiments were performed on Nikon Eclipse Ci-S fluorescence microscope (Nikon, Tokyo, Japan) with 1000× magnification [23].

Confocal laser microscopy

For imaging, M-HeLa and HuTu 80 cells were incubated for 24 hours with F-127-**1** and F-127-**2** NPs at a final concentration 20 µM and 25 µM, respectively. Then cells were washed twice with DPBS and incubated for 20 minutes with MitoTracker Green (50 ng mL^–1^, Invitrogen, USA) and LysoTracker Deep Red (50 nM, Molecular Probes, USA). For microscopic observation and image acquisition Leica TCS SP5 confocal microscope (Leica, Germany) equipped with a set of lasers with a wavelength range from ultra-violet to red was used. Mitotracker Green FM was excited at 488 nm and the fluorescence emission was collected from 500 to 540 nm. LysoTracker was excited at 633 nm and the fluorescence emission was collected from 650 to 680 nm. Signal from samples was excited using multiphoton laser at 350 nm and the fluorescence emission was collected from 400 to 500 nm [26, 27].

Co-localization was analyzed using Las AF software (Leica Biosystems, Germany). The Pearson's correlation coefficient was calculated for each captured field of vision (n = 50), at least 50 cells were taken into account for each field. Data are expressed as means ± SE. The significance of differences of data was analyzed with Student's test. Differences were regarded to be statistically significant at P < 0.05 [26, 27].

**References**

1. Armarego WLF, Chai CLL. Purification of Laboratory Chemicals. Elsevier;2013. <https://doi.org/10.1016/C2009-0-64000-9>.
2. Redmore D. Phosphorus derivatives of nitrogen heterocycles. 2. Pyridinephosphonic acid derivatives. J Org Chem. 1970;35:4114–17. <https://doi.org/10.1021/jo00837a619>.
3. Strelnik ID, Musina EI, Ignatieva SN, Balueva AS, Gerasimova TP, Katsyuba SA, Krivolapov DB, Dobrynin AB, Bannwarth C, Grimme S, Kolesnikov IE, Karasik AA, Sinyashin OG. Pyridyl Containing 1, 5‐Diaza‐3, 7‐diphosphacyclooctanes as Bridging Ligands for Dinuclear Copper (I) Complexes. Z Anorg Allg Chem. 2017;643:895–902. <https://doi.org/10.1002/zaac.201700049>.
4. Strelnik ID, Dayanova IR, Kolesnikov IE, Fayzullin RR, Litvinov IA, Samigullina AI, Gerasimova TP, Katsyuba SA, Musina EI, Karasik AA. The assembly of unique hexanuclear copper (I) complexes with effective white luminescence. Inorg Chem. 2019;58:1048–57. <https://doi.org/10.1021/acs.inorgchem.8b01862>.
5. Faizullin BA, Elistratova JG, Strelnik ID, Akhmadgaleev KD, Gubaidullin AT, Kholin KV, Nizameev IR, Babaev VM, Amerhanova SK, Voloshina AD, Gerasimova TP, Karasik AA, Sinyashin OG, Mustafina AR. Luminescent Water-Dispersible Nanoparticles Engineered from Copper (I) Halide Cluster Core and P, N-Ligand with an Optimal Balance between Stability and ROS Generation. Inorganics. 2023;11:141. <https://doi.org/10.3390/inorganics11040141>.
6. Delgado AV, Gonzalez-Caballero F, Hunter RJ, Koopal LK, Lyklema J. Measurement and interpretation of electro-kinetic phenomena. J Colloid Interface Sci. 2007;309:194–224. <https://doi.org/10.1016/j.jcis.2006.12.075>.
7. Faizullin B, Dayanova I, Strelnik I, Kholin K, Nizameev I, Gubaidullin A, Voloshina A, Gerasimova T, Kashnik I, Brylev K, Sibgatullina G, Samigullin D, Petrov K, Musina E, Karasik A, Mustafina A. pH-Driven Intracellular Nano-to-Molecular Disassembly of Heterometallic [Au2L2]{Re6Q8} Colloids (L= PNNP Ligand; Q= S2− or Se2−). Nanomaterials. 2022;12:3229. <https://doi.org/10.3390/nano12183229>.
8. Khrizanforov MN, Arkhipova DM, Shekurov RP, Gerasimova TP, Ermolaev VV, Islamov DR, Miluykov VA, Kataeva ON, Khrizanforova VV, Sinyashin OG, Budnikova YH. Novel paste electrodes based on phosphonium salt room temperature ionic liquids for studying the redox properties of insoluble compounds. J Solid State Electrochem. 2015;19:2883–90. <https://doi.org/10.1007/s10008-015-2901-0>.
9. Fazleeva RR, Nasretdinova GR, Osin YN, Samigullina AI, Gubaidullin AT, Yanilkin VV. An Effective Producing Method of Nanocomposites of Ag, Au, and Pd Nanoparticles with Poly (N-Vinylpyrrolidone) and Nanocellulose. Electrocatalysis. 2021;12:225–37. <https://doi.org/10.1007/s12678-021-00645-y>.
10. DIFFRAC Plus Evaluation package EVA, Version 11, User’s Manual, Bruker AXS, Karlsruhe, Germany. 2005. 258 p.
11. APEX2 Version 2.1, SAINTPlus. Data Reduction and Correction Program Version 7.31A, Bruker Advansed X-ray Solutions, BrukerAXS Inc., Madison, Wisconsin, USA, 2006.
12. Sheldrik GM. SADABS, Program for empirical X-ray absorption correction. Bruker-Nonis;1990.
13. Sheldrick GM. SHELXT–Integrated space-group and crystal-structure determination. Acta Crystallogr Sect A: Found Adv. 2015;71:3–8. <https://doi.org/10.1107/S2053273314026370>.
14. Sheldrick GM. Crystal structure refinement with SHELXL. Acta Crystallogr Sect C: Struct Chem. 2015;71:3–8. <https://doi.org/10.1107/S2053229614024218>.
15. Farrugia LJ. WinGX and ORTEP for Windows: an update. J Appl Crystallogr. 2012;45:849–54. <https://doi.org/10.1107/S0021889812029111>.
16. Spek AL. PLATON SQUEEZE: a tool for the calculation of the disordered solvent contribution to the calculated structure factors. Acta Crystallogr Sect C: Struct Chem. 2015;71:9–18. <https://doi.org/10.1107/S2053229614024929>.
17. Dolomanov OV, Bourhis LJ, Gildea RJ, Howard JA, Puschmann H. OLEX2: a complete structure solution, refinement and analysis program. J Appl Crystallogr. 2009;42:339–41. <https://doi.org/10.1107/S0021889808042726>.
18. Spek AL. Structure validation in chemical crystallography. Acta Crystallogr Sect D: Biol Crystallogr. 2009;65:148–55. <https://doi.org/10.1107/S090744490804362X>.
19. Macrae CF, Edgington PR, McCabe P, Pidcock E, Shields GP, Taylor R, Towler M, Streek JVD. Mercury: visualization and analysis of crystal structures. J Appl Crystallogr. 2006;39:453–7. <https://doi.org/10.1107/S002188980600731X>.
20. Elistratova J, Mukhametshina A, Kholin K, Nizameev I, Mikhailov M, Sokolov M, Khairullin R, Miftakhova R, Shammas G, Kadirov M, Petrov K, Rizvanov A, Mustafina A. Interfacial uploading of luminescent hexamolybdenum cluster units onto amino-decorated silica nanoparticles as new design of nanomaterial for cellular imaging and photodynamic therapy. J. Colloid Interface Sci. 2019;538:387–96. <https://doi.org/10.1016/j.jcis.2018.12.013>.
21. MTT Assay Protocol for Cell Viability and Proliferation. Available online: <https://www.sigmaaldrich.com/RU/en/technical-documents/protocol/cell-culture-and-cell-culture-analysis/cell-counting-and-health-analysis/cell-proliferation-kit-i-mtt>.
22. Quest Graph™ IC_50_ Calculator, AAT Bioquest, Inc. Available online: <https://www.aatbio.com/tools/ic50-calculator>. Accessed 12 May 2023.
23. Kuznetsova DA, Gabdrakhmanov DR, Gaynanova GA, Vasileva LA, Kuznetsov DM, Lukashenko SS, Voloshina AD, Sapunova AS, Nizameev IR, Sibgatullina GV, Samigullin DV, Kadirov MK, Petrov KA, Zakharova LY. Novel biocompatible liposomal formulations for encapsulation of hydrophilic drugs–Chloramphenicol and cisplatin. Colloids Surf., A. 2021;610:125673. <https://doi.org/10.1016/j.colsurfa.2020.125673>.
24. Alexa Fluor®488 Annexin V/Dead Cell Apoptosis Kit, Thermo Fisher Scientific Inc. Available online: <https://www.thermofisher.com/ru/ru/home/references/protocols/cell-and-tissue-analysis/flow-cytometry-protocol/apoptosis/alexa-fluor-488-annexin-v-dead-cell-apoptosis-kit.html#comergent_product_list_61758>.
25. CellROX® Oxidative Stress Reagents, Life Technologies Corporation. Revised: 6–May–2012/ MP 10422. Available online: <https://tools.thermofisher.com/content/sfs/manuals/mp10422.pdf>
26. Faizullin BA, Strelnik ID, Dayanova IR, Gerasimova TP, Kholin KV, Nizameev IR, Voloshina AD, Gubaidullin AT, Fedosimova SV, Mikhailov MA, Sokolov MN, Sibgatullina GV, Samigullin DV, Petrov KA, Karasik AA, Mustafina AR. Structure impact on photodynamic therapy and cellular contrasting functions of colloids constructed from dimeric Au (I) complex and hexamolybdenum clusters. Mater. Sci. Eng., C. 2021;128:112355. <https://doi.org/10.1016/j.msec.2021.112355>.
27. Kuznetsova DA, Gaynanova GA, Vasileva LA, Sibgatullina GV, Samigullin DV, Sapunova AS, Voloshina AD, Galkina IV, Petrov KA, Zakharova LY. Mitochondria-targeted cationic liposomes modified with alkyltriphenylphosphonium bromides loaded with hydrophilic drugs: Preparation, cytotoxicity and colocalization assay. J. Mater. Chem. B. 2019;7:7351–62. <https://doi.org/10.1039/C9TB01853K>.

**Fig.** **S1.** NMR ^31^P spectrum of ligand **L1** measured in DMF-d_7_.

**Fig.** **S2.** NMR ^31^P spectrum of ligand **L2** measured in CDCl_3_.

**Fig.** **S3.** NMR ^31^P spectrum of complex **1** measured in acetone-d_6_.

**Fig. S4.** NMR ^31^P spectrum of complex **2** measured in CD_3_CN.

**Fig.** **S5.** NMR ^31^P spectrum of complex **3** measured in CD_3_CN.

**Fig.** **S6.** NMR ^31^P spectrum of complex **4** measured in CD_3_CN.


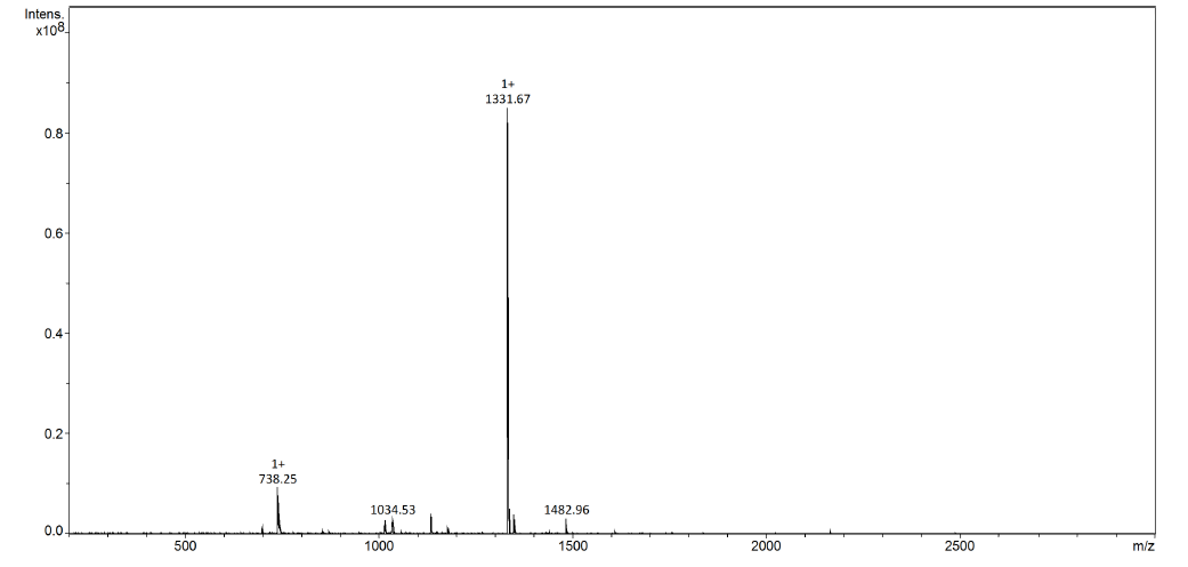


**Fig.** **S7.** ESI mass-spectrum of complex **1** measured in DMF.


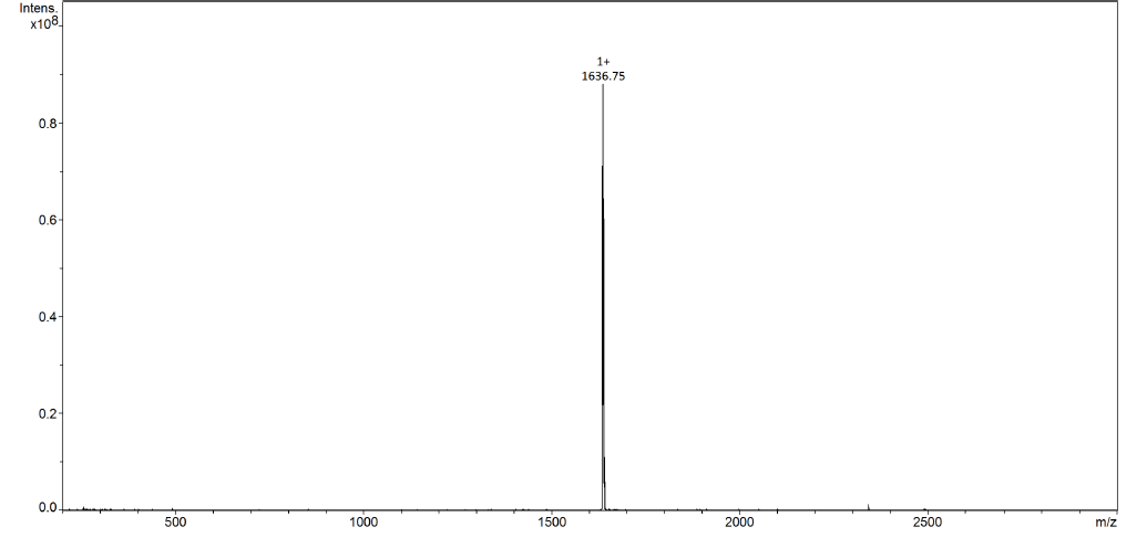


**Fig. S8.** ESI mass-spectrum of complex **2** measured in CH_3_CN.

**
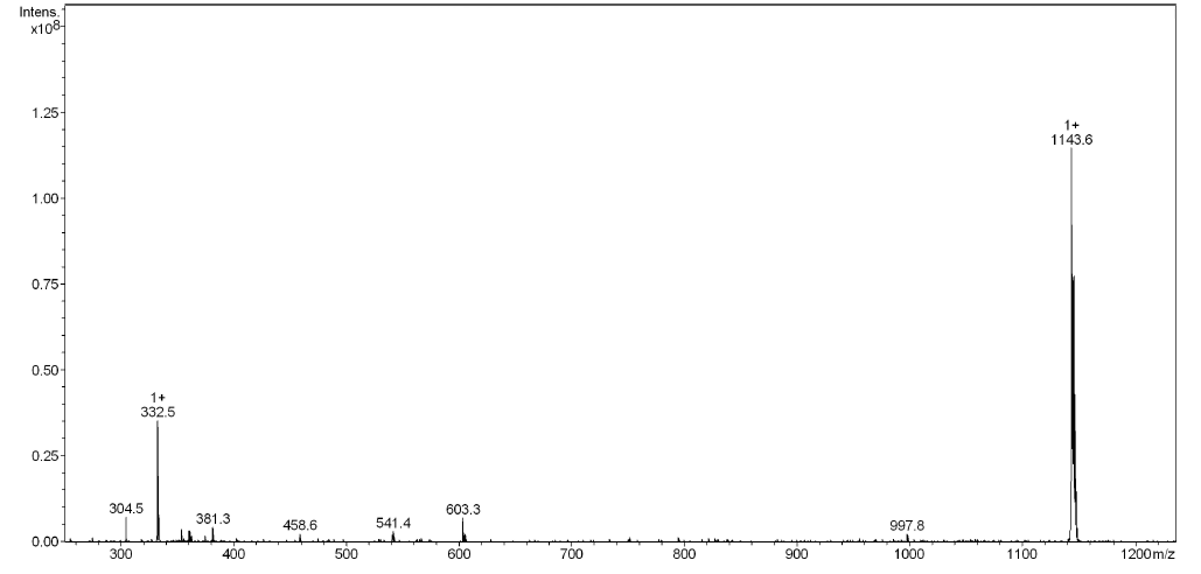
**

**Fig. S9.** ESI mass-spectrum of complex **3** measured in CH_3_CN.

**
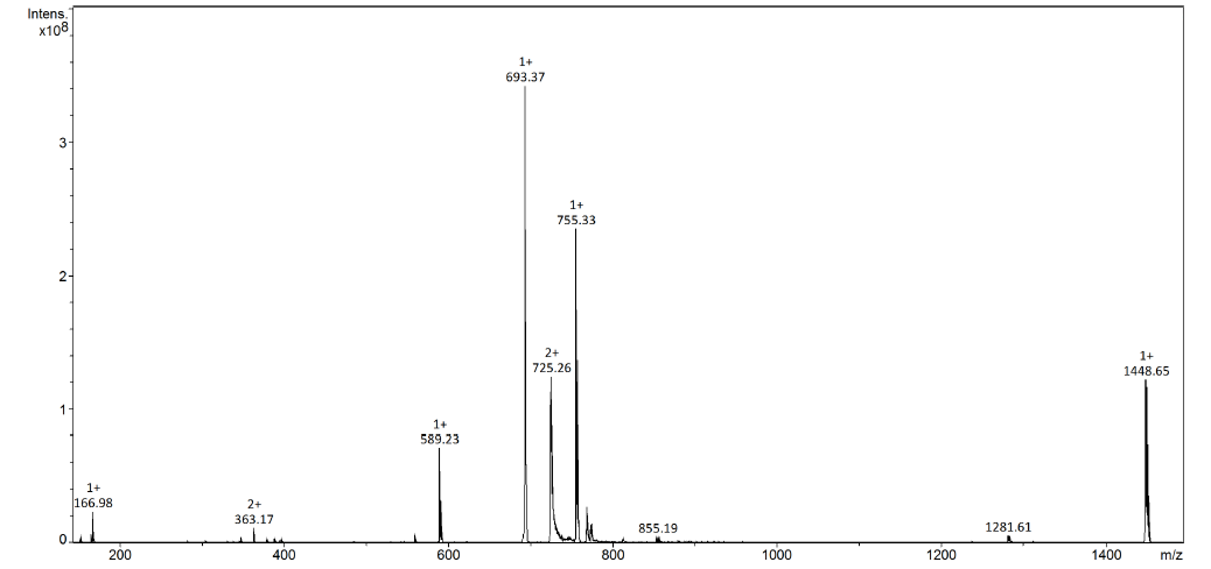
**

**Fig. S10.** ESI mass-spectrum of complex **4** measured in CH_3_CN.

**
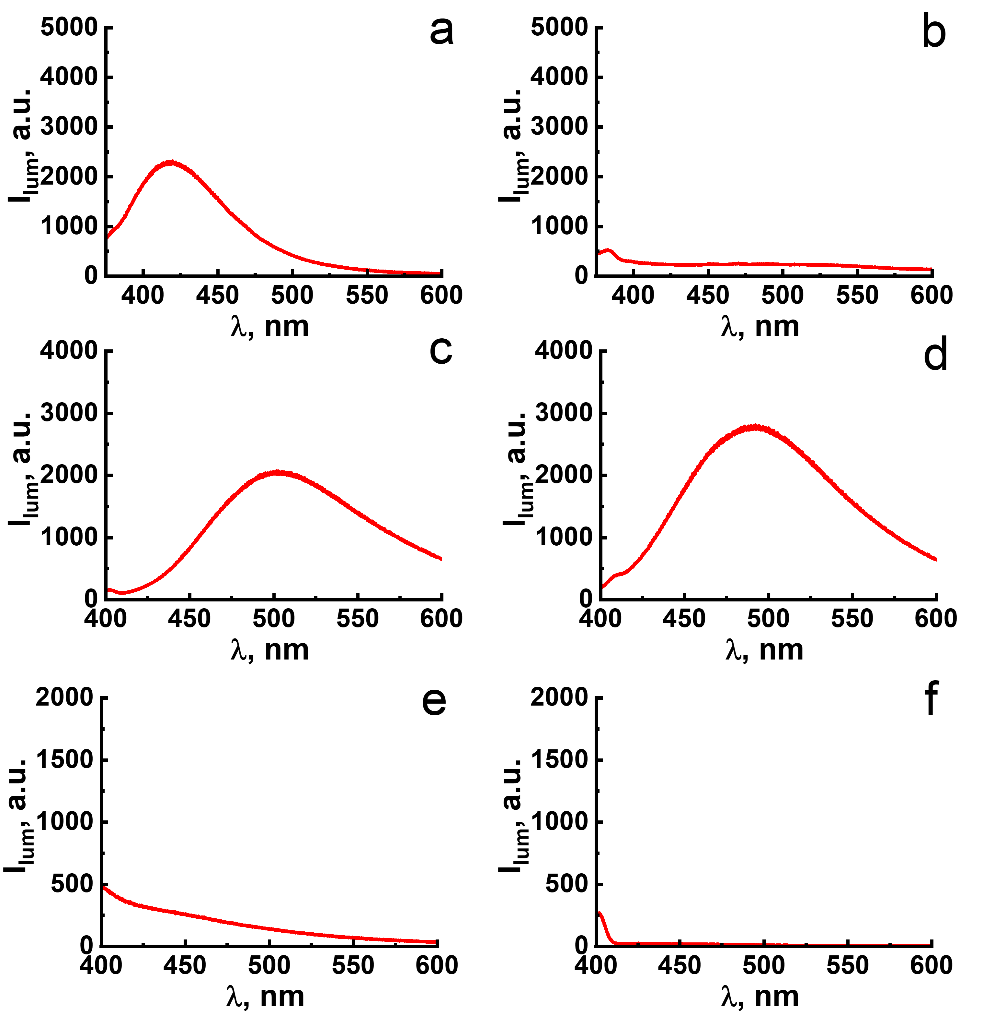
**

**Fig. S11.** Emission spectra of F-127-**3** (a) and F-127-**4** (b) nanoparticles and DMF solutions of complexes **1**-**4** (c-f). λ_ex_ = 340 nm.

**Table S1.** Average (*d*_av_) and evaluated through the size distribution by number (*d*_num_) diameter values, polydispersity indices (PDI) and electrokinetic potentials (ζ) of F-127-**3**(**4**) nanoparticles.

|  | ***d*_av_, nm** | ***d*_num_, nm** | **PDI** | **ζ, mV** |
| --- | --- | --- | --- | --- |
| F-127-**3** | – | – | 1 | – |
| F-127-**4** | 203±3 | 193±5 | 0.081±0.020 | +22±12 |

**Table S2.** ICP-OES data and calculated Cu:P molar ratio in F-127-**1** and F-127-**2** NPs.

|  | **Elements content mg/L, ±10%** | | |
| --- | --- | --- | --- |
|  | **Cu (324.754 nm)** | **P (178.284 nm)** | **Cu:P ratio** |
| F-127-**1** | 0.309 | 0.667 | 1:4.41 |
| F-127-**2** | 0.411 | 1.00 | 1:4.99 |

**
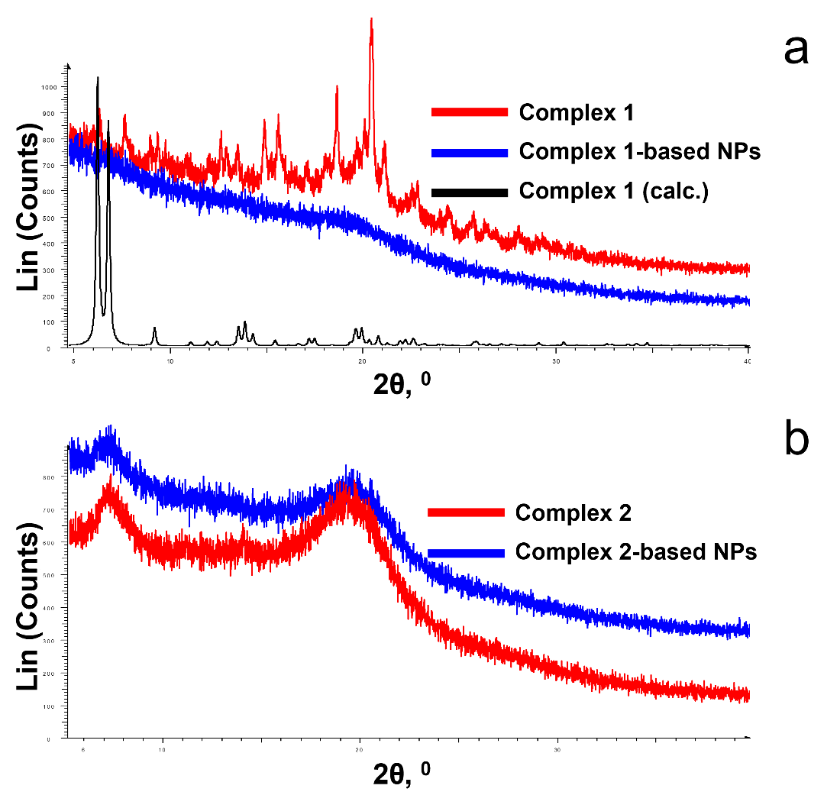
**

**Fig. S12.** Experimental diffractograms of complexes **1** and **2** and corresponding complexes-based nanoparticles and calculated powder diffraction pattern for complex **1**. The curves are shifted along intensity axis for clarity.

**
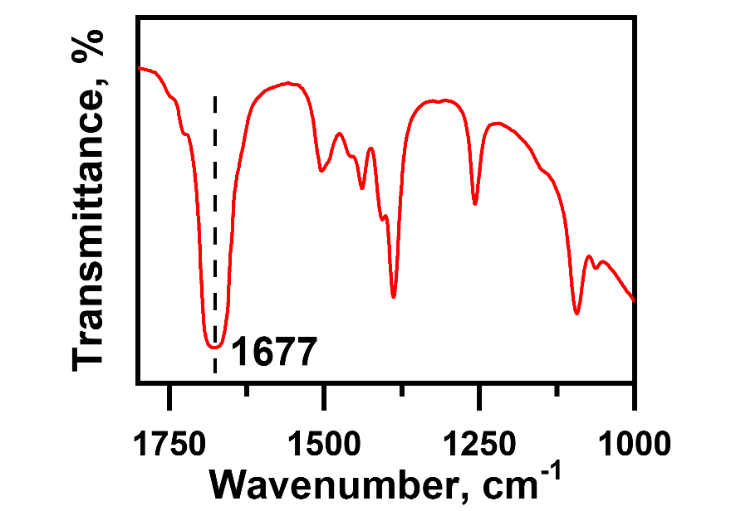
**

**Fig. S13.** FTIR spectrum of DMF.

**
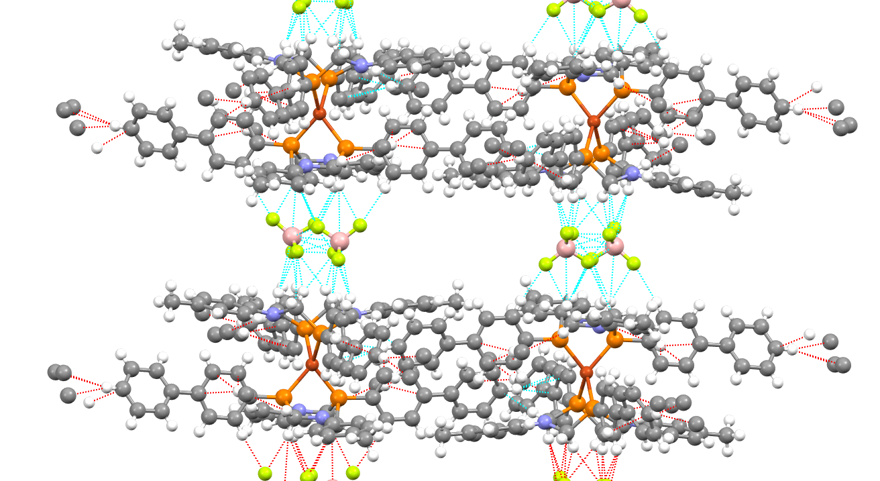
**

**Fig. S14.** Fragment of complex **1** crystal packing. Dashed lines correspond to short contacts (< sum vdW radii).


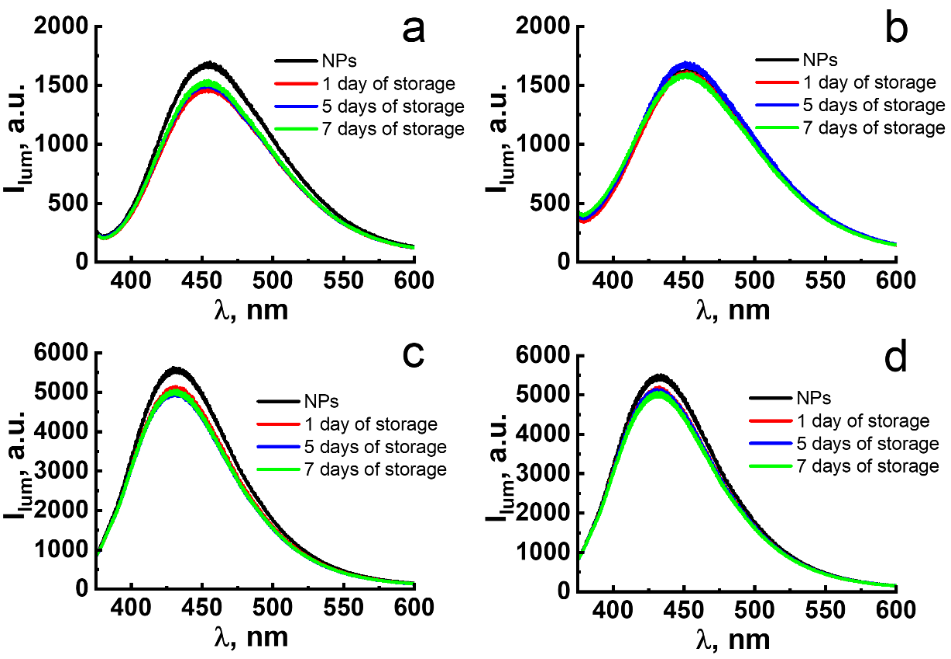


**Fig. S15.** Luminescence spectra of buffered (a, c – pH = 4; b, d – pH = 7) solutions of F-127-**1** (a, b) and F-127-**2** (c, d) NPs registered within 7 days.

**Table S3.** Average (*d*_av_) and evaluated through the size distribution by number (*d*_num_) and volume (*d*_vol_) diameters, polydispersity indices (PDI) and electrokinetic potentials (ζ) of F-127-**1**(**2**) measured after 1 day of storage in phosphate buffer (pH 7.0) without/in the presence of GSH or H_2_O_2_.

|  | ***d*_av_, nm** | ***d*_num_, nm** | ***d*_vol_, nm** | **PDI** | **ζ, mV** |
| --- | --- | --- | --- | --- | --- |
| F-127-**1** | 144±1 | 119±9 | 143±3 | 0.102±0.006 | –4±6 |
| F-127-**1**+H_2_O_2_ | 127±2 | 93±27 | 119±10 | 0.129±0.011 | –3±7 |
| F-127-**1**+GSH | 146±1 | 98±26 | 436±276 | 0.229±0.014 | –6±12 |
| F-127-**2** | 156±1 | 135±6 | 160±4 | 0.091±0.014 | –1±9 |
| F-127-**2**+H_2_O_2_ | 131±1 | 89±19 | 125±6 | 0.099±0.022 | –2±7 |
| F-127-**2**+GSH | 133±1 | 107±5 | 180±71 | 0.104±0.016 | –5±15 |

**Table S4.** Average (*d*_av_) and evaluated through the size distribution by number (*d*_num_) diameters, polydispersity indices (PDI) and electrokinetic potentials (ζ) of F-127-**1**(**2**) NPs measured while storage in pure water and buffered solutions, after heating up to 40 ^0^C and in the presence of H_2_O_2_ and GSH.

|  | ***d*_av_, nm** | ***d*_num_, nm** | **PDI** | **ζ, mV** |
| --- | --- | --- | --- | --- |
| **Storage for 1 month in H_2_O:** | | | | |
| F-127-**1** | 119±1 | 100±7 | 0.096±0.029 | +30±7 |
| F-127-**2** | 132±1 | 117±4 | 0.077±0.013 | +35±6 |
| **Storage for 1 week at different pH:** | | | | |
| F-127-**1*** | 139±1 | 125±6 | 0.079±0.022 | +16±3 |
| F-127-**1**** | 146±2 | 126±6 | 0.139±0.042 | –3±6 |
| F-127-**2*** | 153±2 | 137±9 | 0.070±0.020 | +15±5 |
| F-127-**2**** | 161±3 | 141±7 | 0.105±0.008 | –3±5 |
| **After heating up to 40C:** | | | | |
| F-127-**1** | 125±1 | 111±2 | 0.095±0.017 | +7±8 |
| F-127-**2** | 131±2 | 110±4 | 0.105±0.011 | +20±7 |
| **Storage for 1 day in the presence of H_2_O_2_:** | | | | |
| F-127-**1**** | 127±2 | 93±27 | 0.129±0.011 | –3±7 |
| F-127-**2**** | 131±1 | 89±19 | 0.099±0.022 | –2±7 |

* pH 4.0

** pH 7.0

**
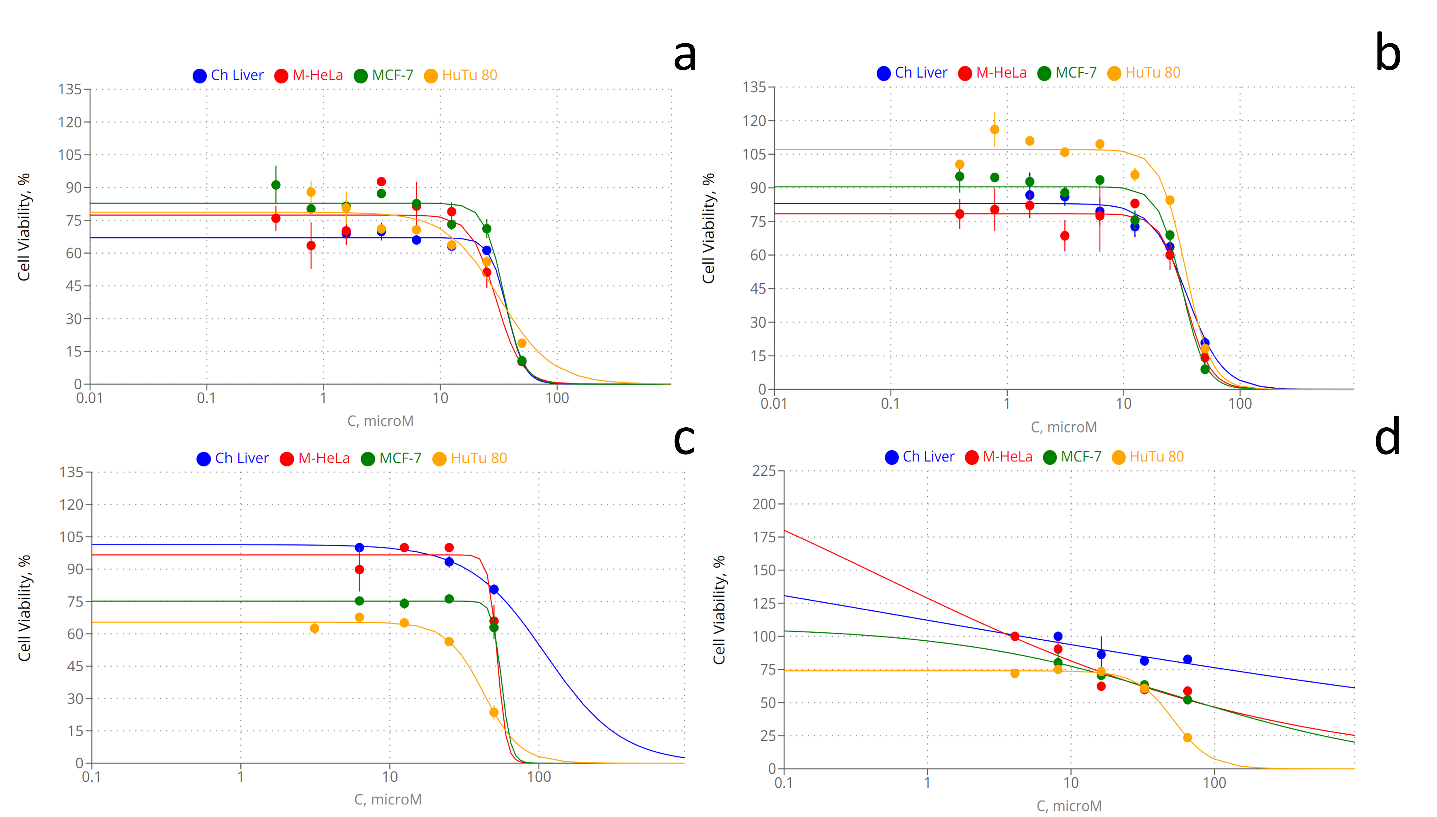
**

**Fig. S16.** Viability of Chang Liver, M-HeLa, MCF-7 and HuTu 80 cells incubated with different concentrations of complexes **1** (a), **2** (b) and F-127-**1** (c) and F-127-**2** (d) NPs. The error bars represent standard deviation of the mean values.

**
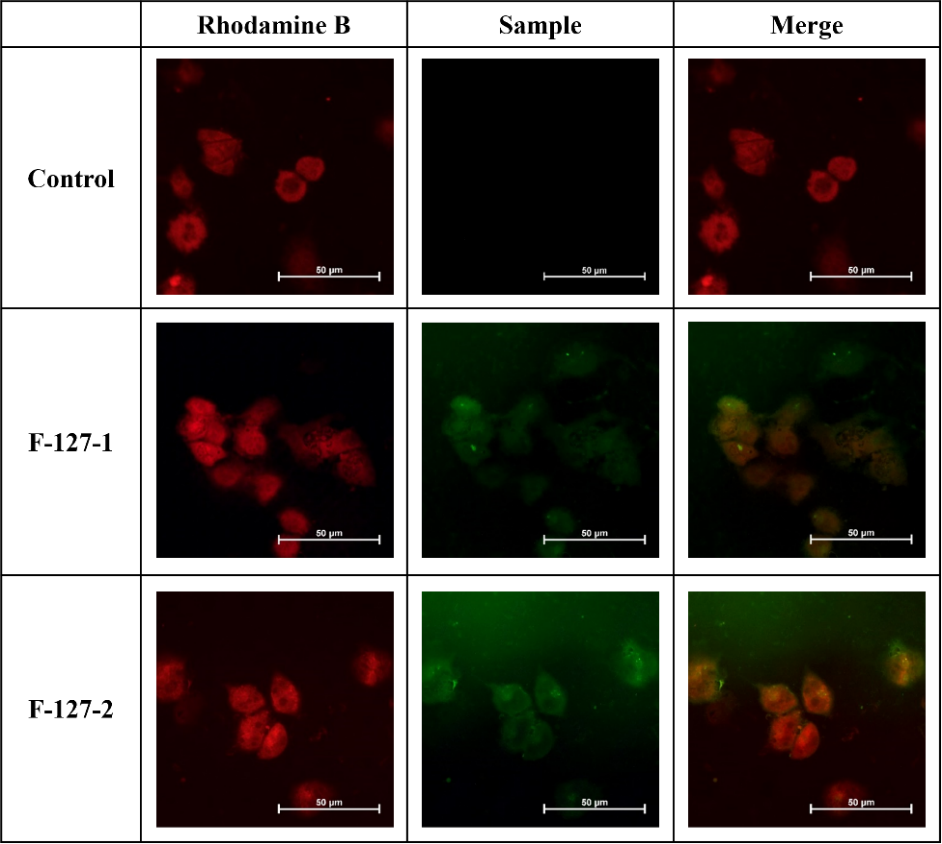
**

**Fig. S17.** Fluorescence microscopy images of HuTu 80 cell line co-incubated by F-127-**1** and F-127-**2** NPs and Rhodamine B.

**
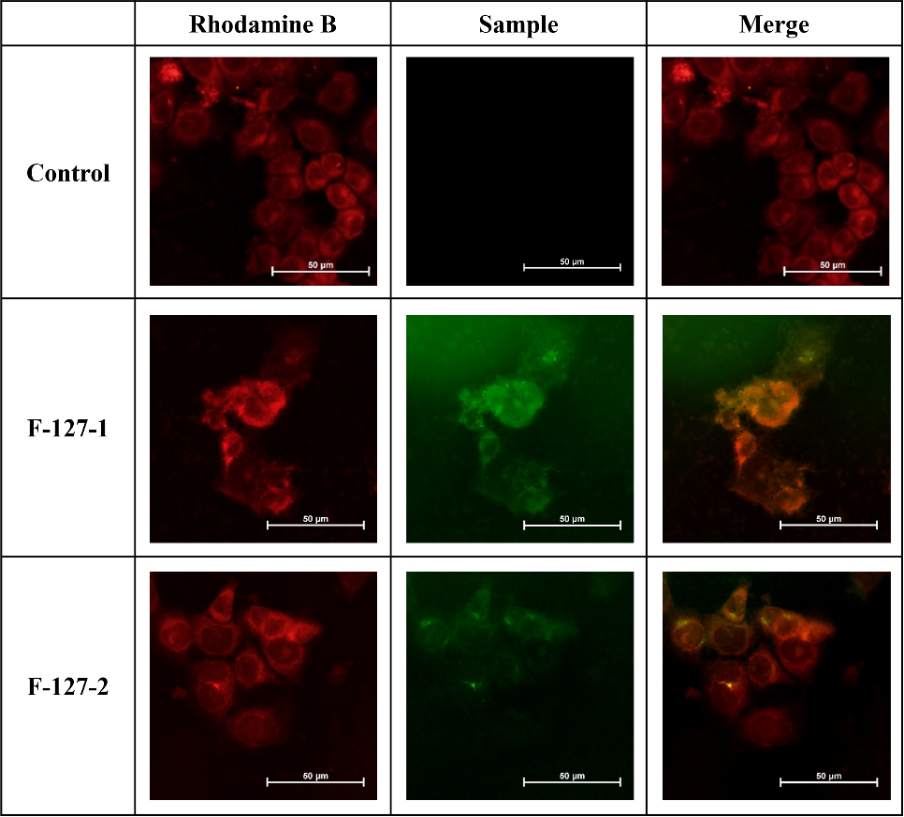
**

**Fig. S18.** Fluorescence microscopy images of M-HeLa cell line co-incubated by F-127-**1** and F-127-**2** NPs and Rhodamine B.

**
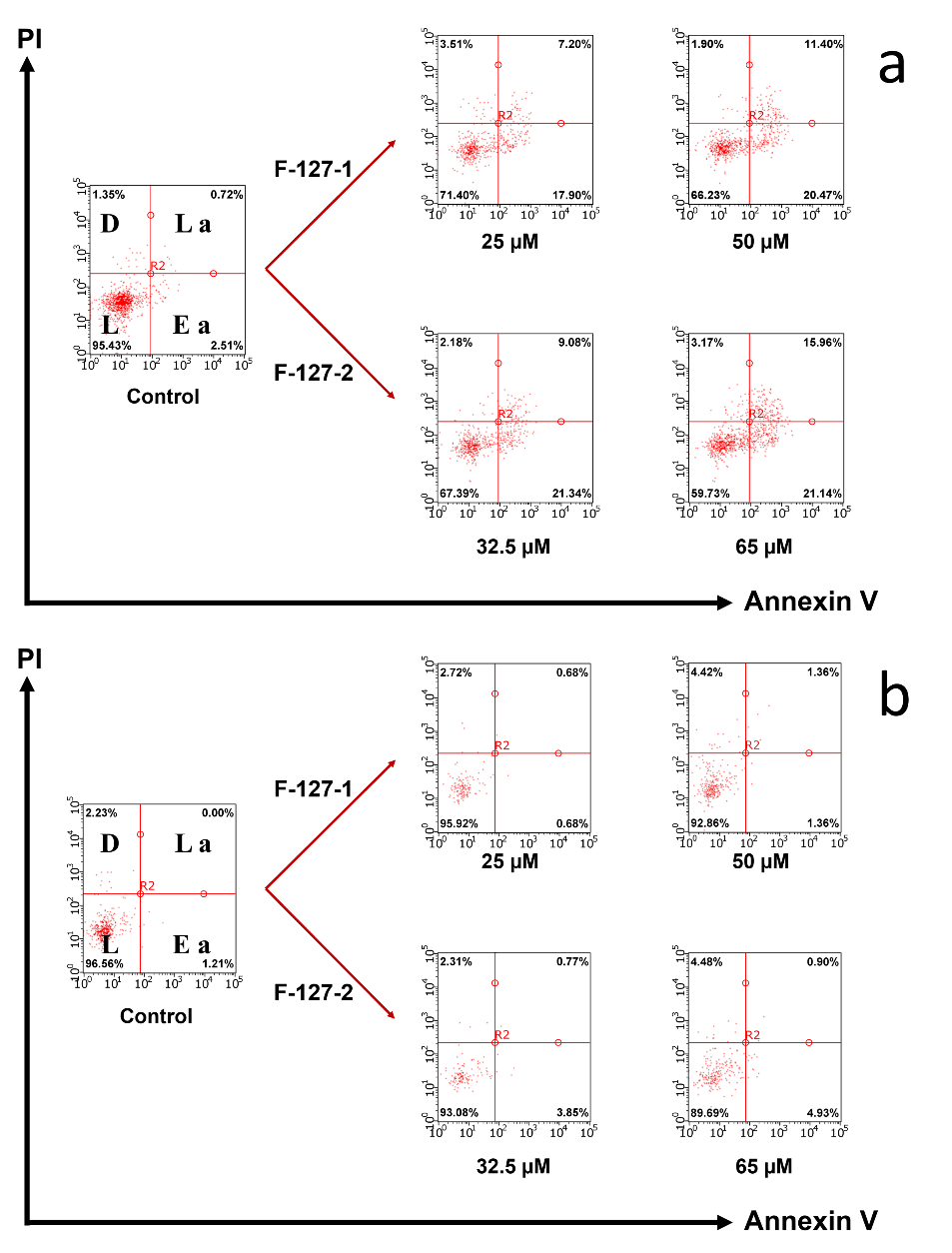
**

**Fig. S19.** Two-parameter plots from flow cytometry analysis of HuTu 80 (a) and M-HeLa (b) cells treated with different concentrations of F-127-**1** and F-127-**2** NPs after Annexin V and PI staining. The values are presented as the mean ± SD (n = 3).

**
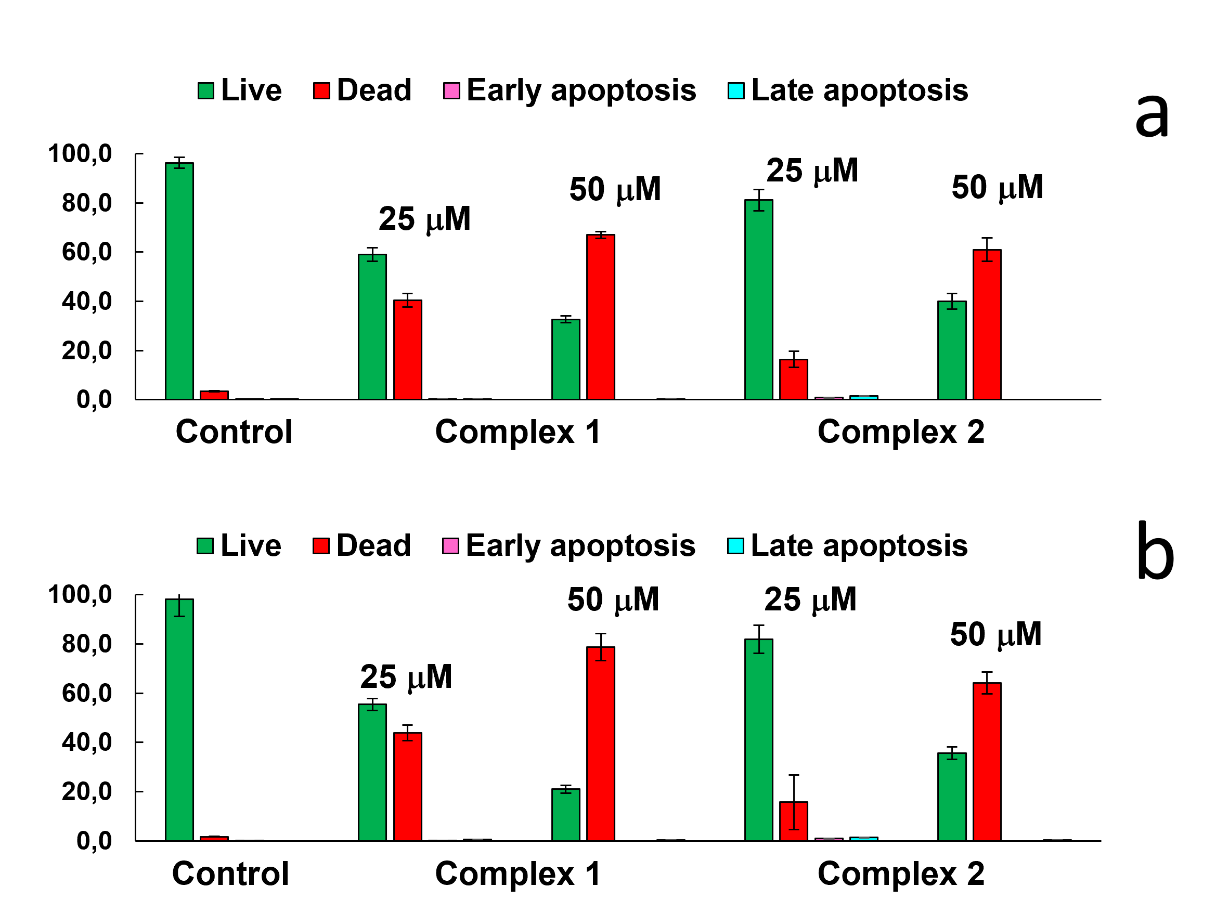
**

**Fig. S20.** Flow cytometry analysis of HuTu 80 (a) and M-HeLa (b) cells treated with different concentrations of complexes **1** and **2** after Annexin V and PI staining. The values are presented as the mean ± SD (n = 3).
